# Assessing the safety of physical rehabilitation in critically ill patients: a Delphi study

**DOI:** 10.1186/s13054-024-04919-x

**Published:** 2024-04-30

**Authors:** Huw R. Woodbridge, Christopher J. McCarthy, Mandy Jones, Matthew Willis, David B. Antcliffe, Caroline M. Alexander, Anthony C. Gordon

**Affiliations:** 1 Imperial College Healthcare NHS Trust, London, UK; 2 Department of Surgery and Cancer, Imperial College London, London, UK; 3 Manchester Metropolitan University, Manchester, UK; 4 Brunel University London, London, UK

**Keywords:** Mobilisation, Adverse events, Vasoactive drugs, Intensive care unit

## Abstract

**Background:**

Physical rehabilitation of critically ill patients is implemented to improve physical outcomes from an intensive care stay. However, before rehabilitation is implemented, a risk assessment is essential, based on robust safety data. To develop this information, a uniform definition of relevant adverse events is required. The assessment of cardiovascular stability is particularly relevant before physical activity as there is uncertainty over when it is safe to start rehabilitation with patients receiving vasoactive drugs.

**Methods:**

A three-stage Delphi study was carried out to (a) define adverse events for a general ICU cohort, and (b) to define which risks should be assessed before physical rehabilitation of patients receiving vasoactive drugs. An international group of intensive care clinicians and clinician researchers took part. Former ICU patients and their family members/carers were involved in generating consensus for the definition of adverse events. Round one was an open round where participants gave their suggestions of what to include. In round two, participants rated their agreements with these suggestions using a five-point Likert scale; a 70% consensus agreement threshold was used. Round three was used to re-rate suggestions that had not reached consensus, whilst viewing anonymous feedback of participant ratings from round two.

**Results:**

Twenty-four multi-professional ICU clinicians and clinician researchers from 10 countries across five continents were recruited. Average duration of ICU experience was 18 years (standard deviation 8) and 61% had publications related to ICU rehabilitation. For the adverse event definition, five former ICU patients and one patient relative were recruited. The Delphi process had a 97% response rate. Firstly, 54 adverse events reached consensus; an adverse event tool was created and informed by these events. Secondly, 50 risk factors requiring assessment before physical rehabilitation of patients receiving vasoactive drugs reached consensus. A second tool was created, informed by these suggestions.

**Conclusions:**

The adverse event tool can be used in studies of physical rehabilitation to ensure uniform measurement of safety. The risk assessment tool can be used to inform clinical practise when risk assessing when to start rehabilitation with patients receiving vasoactive drugs.

*Trial registration* This study protocol was retrospectively registered on https://www.researchregistry.com/ (researchregistry2991).

**Supplementary Information:**

The online version contains supplementary material available at 10.1186/s13054-024-04919-x.

## Background

Physical rehabilitation or mobilisation of patients whilst they are admitted to an intensive care unit (ICU) is implemented to reduce the physical complications of critical illness and to improve patient outcomes [1, 2]. Physical rehabilitation is associated with low adverse event rates [3], however recent data have suggested that a higher rehabilitation dose delivered at an early time point may be less safe with no added benefit on outcomes [4]. Clearly, detailed risk assessment is required to judge when it is safe to start rehabilitation [5, 6].

The interpretation of ICU physical rehabilitation safety data is limited by the variation in how studies define their adverse event outcomes [3]. Key differences include which physiological variables are included and what constitutes an unsafe change in physiological variables such as blood pressure [7–12]. Previous efforts at reaching consensus on an adverse event tool [3, 13] have not seen uniform adoption in studies [4, 14–18] and did not include patient or caregiver opinions so did not capture events that were important to service-users, which may have limited tool uptake. An internationally agreed uniform adverse event definition would also allow future studies to be compared and more readily combined for greater power [3, 19]. Designing an adverse event definition using a Delphi process, facilitates a methodical consensus among key stakeholders whilst anonymising opinion to prevent the process being unduly influenced by prominent participants [20, 21].

A key safety consideration is whether critically ill patients have the cardiovascular capacity to withstand physical activity, particularly if they are receiving vasoactive drugs [5, 22, 23]. Cardiovascular instability accounts for a substantial number of reported adverse events during rehabilitation [3, 8] and is frequently cited as a barrier to starting rehabilitation [24–31]. Vasoactive drugs are a key consideration in assessing cardiovascular stability as they are prescribed to improve cardiac output and elevate blood pressure [32, 33], which are challenged through rehabilitation activities [3, 6]. However, there is a lack of agreement between practice guidelines and the ‘rehabilitation readiness’ criteria used in studies, over when it is safe to start rehabilitation and how to assess risk with patients receiving vasoactive drugs [5, 18, 34, 35], which may lead to variations in practice [36, 37]. Further consensus is therefore required to guide clinicians on how to risk assess the implementation of rehabilitation with patients receiving vasoactive drugs. This would need to include considerations such as drug dose and cardiovascular stability [5].

This study had two aims:

1. To develop an expert, multi-professional clinician and patient consensus agreement on the definition of an adverse event occurring whilst an adult patient receives physical rehabilitation in an ICU. The defined adverse events will be included in an adverse event tool.
2. To determine an expert, multi-professional clinician consensus on the defining characteristics of adult ICU patients receiving vasoactive drugs, who have a low or a higher risk of adverse events when receiving physical rehabilitation and the characteristics of patients in whom rehabilitation is contraindicated.

## Methods

In this study, an international, three-stage Delphi process was used to reach consensus on (a) an adverse event definition for rehabilitation for general ICU patients and (b) a risk assessment tool for rehabilitation for patients receiving vasoactive drugs. The Delphi process consisted of repeated rounds of questionnaires. Round one was an open round in which participants were able to give their suggestions for what to include in the two tools. Following this, participants rated their agreement with these suggestions. Consensus was facilitated in round three when participants re-rated their opinion after viewing anonymous feedback of the opinion of other participants given in round two [20]. The Delphi method provides the advantage of this anonymous feedback ensuring that participants are not unduly influenced by particular higher profile participants or more expressive personalities [21]. The procedures outlined below were based on those set out by Keeney, Hasson and McKenna [38]. Ethical approval was gained for the study (London—Camberwell St Giles Research Ethics Committee, 17/LO/0830) and informed consent received from all participants. This study is reported based upon Conducting and Reporting Delphi Studies (CREDES) criteria [39].

### Participants

Purposive sampling [20] was used to select a group of ICU clinicians and clinician researchers from a range of different continents and professions to gain a range of relevant views for forming an adverse event tool and a clinical risk assessment tool. Clinicians were approached through contact details of the corresponding authors of papers gathered after a background literature search of the topics under consideration here [40, 41]. These clinicians were also asked to forward study information to their relevant contacts [42]. Participants were also sourced through the international networks of the authors [43, 44]. Inclusion criteria for the clinician group were that participants be medical doctors, nurses and physiotherapists (or physical therapists) working at a grade equivalent to a clinical/team leader in their professional group on their ICU. In addition, clinicians must have been personally involved in a clinical decision about mobilising an ICU patient within the previous year.

For the adverse event tool only, a range of former ICU patients and their relatives or carers were purposively sampled. They were identified by their response to an email circulated via a national ICU patient support group or to an in-person study advert at a local hospital group. Patients were then selected by the principles of purposeful sampling (to maximise patient/carer experience from a range of ICU settings) and if they met the inclusion/exclusion criteria. This participant group were included if they had any experience of the ICU environment. All potential participants were excluded if they were unable to participate using email or post (if based in the UK), if they felt unable to read and write in detail in English and if their age was less than 18 years. Former patients or relatives/carers were excluded if they were unable to give informed consent and if they were unable to participate in an initial meeting in person or via video conferencing software. This meeting was held to provide information about the Delphi methodology and to provide clarity on the research topic to ensure full participation [45]. Patients/relatives were not included in the risk assessment tool process as this related to clinician decision-making and required expert clinical knowledge. No additional participants were recruited after data collection had begun.

The sample size for this study originally aimed for 23 participants, the same number who participated in previous clinical ICU rehabilitation guidance development [5]. However, this was increased to 30 when it became apparent during the study that it would be possible to recruit a greater range of participants from different professional backgrounds and geographical areas. This original sample aim was to include 18 clinicians and five patients or caregivers, which was then increased to 24 clinicians and six patients/caregivers. This provided a comparable proportion of patients and caregivers to other critical care Delphi studies [46, 47]. This final sample size maximised the range of opinion within the resource constraints of the study [45, 48].

### Delphi process

**Fig. 1:**
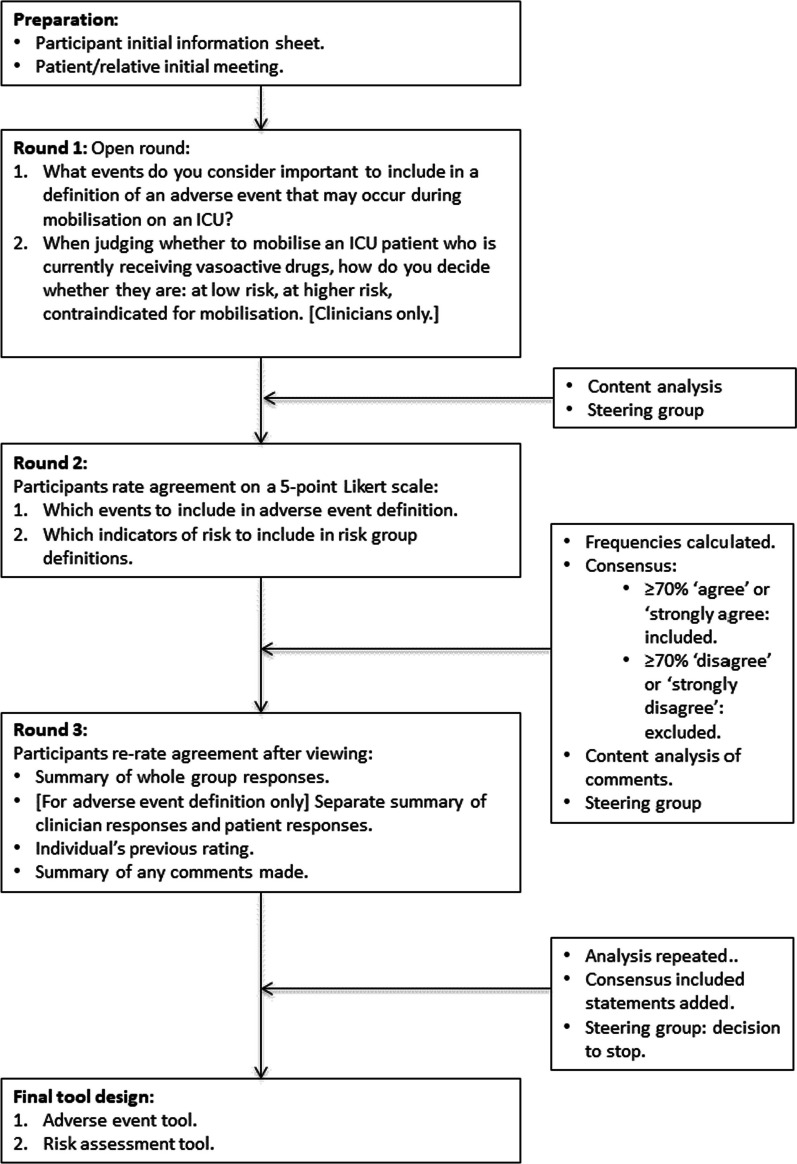
Outline of Delphi process

Three rounds of the Delphi process (Fig. 1) were used to provide enough opportunity to develop consensus whilst maintaining a concise process to maximise response rate [49, 50]. The questionnaires for all three rounds were drafted before the study began, including separate versions with instructions in lay language for patients and relatives [47]. The structure and content of questionnaires for rounds two and three were finalised in conjunction with the steering group, after analysis of the preceding round was complete. (See Additional File 1 for the questionnaires used in each round.) Before being sent to participants, the questionnaires were informally piloted and refined [20] with colleagues of the research team and members of the steering group. Questionnaires were designed in Microsoft Word and mainly distributed via email, with a postal option if requested by patient/relative participants. Response rate was maximised by making efforts to ensure questionnaires were concise and understandable, using email reminders to return completed questionnaires and following-up non responders with a further email then a telephone call [41, 44, 45, 51–53].

### Round 1

Round one was an open round where participants were asked to provide suggestions for a) how to define an adverse event and b) for how to define when a patient receiving vasoactive drugs was at low or higher risk of an adverse event with physical rehabilitation or if rehabilitation was contraindicated. Risk group definitions were based upon previous clinician guidance [5, 54]. Details of how risk was defined and how participant responses were structured [5] can be found in the questionnaires contained in Additional File 1. Demographic information was also recorded and all participants were provided with a supporting information sheet that clarified the scope of the questions asked (see Additional File 1). Whilst the adverse event tool was for a general ICU population for all physical rehabilitation, the risk assessment tool was designed for patients receiving vasoactive drugs, excluding brain injured patients with specific haemodynamic targets and only in relation to rehabilitation related to out of bed activities. Participant responses were summarised using content analysis.

### Round 2

In the round two questionnaire, participants were presented with an amalgamated list of the suggestions provided in round one and were asked to rate whether each adverse event and indicator of risk should be included in the final definitions using a 5-point Likert scale, ranging from strongly disagree to strongly agree. Those items that reached consensus were then removed from the process. Consensus was defined a priori as ≥ 70% [41, 46, 55] of participants in agreement (agreed plus strongly agreed ratings), or, ≥ 70% or more in disagreement (disagreed plus strongly disagreed ratings). There was a large number of suggestions for the three risk group definitions (low, higher and contraindicated). Therefore, rather than voting for the three risk groups separately, the steering group decided it was more practical and concise for participants to rate each indicator of risk once. Participants would then rate if an indicator of risk showed a patient had become higher risk, or if it showed that a patient was contraindicated for mobilisation (see Additional File 1: Round two questionnaire). Furthermore, in round one participants suggested general risk considerations which were rated separately using the Likert scale. Finally, participants were also able to provide comments to explain their ratings [45, 46, 56].

### Round 3

Those items which had not reached consensus were then included in round three. Events that had reached consensus in round two were not reconsidered as the resulting lengthier questionnaires were thought likely to impact response rate [52]. To support decision making, participants re-rated undecided items on the 5-point Likert scale after viewing their previous rating from Round 2, anonymised summary feedback of how the participant group as a whole rated items in the previous round (including mode response), plus a summary of any comments made by participants [42]. In addition, for the adverse event definition, a summary of how the clinician group and patient group voted were listed separately to ensure the patient opinion was made clear [46]. Participants were also informed of items that reached consensus in round two. For the vasoactive drug risk tool, of those items left undecided by the end of round three, the steering group decided to include indicators where the sum of participant ratings for ‘agree’ plus ‘strongly agree’ (for higher risk) *plus* ‘contraindicated’ were ≥ 70%.

### Adverse event and risk assessment tool design

All items that reached consensus for agreement by participants in the Delphi process were included in the final tools. To ensure a lack of duplication, very similar items were combined and the tools were refined by study authors. Finally, the tools were informally tested and then refined in response to feedback from our hospital ICU clinicians and clinical academic colleagues from a range of professional backgrounds. These were not participants in the Delphi process. To achieve this, development of the final adverse event tool was carried out by study authors from a physiotherapy and physician background (HRW, MJ, CMA and ACG) and firstly involved amalgamating the events that reached consensus for inclusion into a concise form. Events were grouped together into categories and any general statements were included at the end to cover anything not already captured by more precise statements. A convenience sample of nineteen clinical physiotherapy colleagues from our hospital assessed and gave feedback on the draft tool, leading to wording changes to improve clarity. These were all the physiotherapists being trained to use the tool in preparation for a future observational study of physical rehabilitation on ICU (NCT03869541). The risk assessment tool development occurred with four study authors from a physician and physiotherapy background (HRW, DBA, CMA and ACG) and consisted of amalgamating similar indicators of risk and making semantic changes to wording to improve clarity. The tool was then tested with a convenience sample of five clinical and clinical academic colleagues from our hospital including two clinical academic lead ICU physicians, a clinical academic physiotherapist, and a senior ICU nurse and physiotherapist. They were chosen as they were experienced colleagues representingthe breadth of professions who participated in the Delphi process and included clinicians who would be involved in clinical risk assessment decision making about initiating rehabilitation with patients receiving vasoactive drugs.

### Steering committee

The study steering group included members with subject and methods expertise (HRW, CJM, MJ, CMA, ACG), plus a patient representative. They helped to pilot and refine questionnaires, including finalising round two and three after analysis of preceding rounds. Furthermore, the group were involved in decisions guided by a priori criteria: Firstly, deciding which adverse events were not relevant to list for patients to rate involved selecting those including physiological variables which required judging different numbers or values. Secondly, due to concern that patient participants could be effectively outvoted by clinicians, the steering group highlighted differences of opinion between patients/relatives and clinicians for consensus events and decided which should be reassessed in round three. Finally, the steering group decided whether to end the study after a third or fourth round, based on a priori criteria of adequate consensus reached to form the adverse event and risk assessment tools. Additionally, the steering group reviewed analysis after each round and made pragmatic decisions over the construction of the next round and highlighting participant views, which are set out elsewhere in the methods and results.

### Data analysis

IBM SPSS Statistics was used to summarise participant demographics and Likert ratings. Normality of continuous data was tested for using the Shapiro–Wilk test. If a patient/carer rated an item as ‘unable to comment’, or if an item was not rated by patients/carers, it was excluded from percentage consensus calculation. Qualitative inductive content analysis [57, 58], assisted by NVivo 11 software (QSR International) was used to analyse and amalgamate the free-text responses to round one, as well as any comments made in rounds two and three. A second researcher confirmed the results of the content analysis by checking a portion of the data and queries over unclear statements were resolved with an additional researcher [59].

## Results

**Fig. 2:**
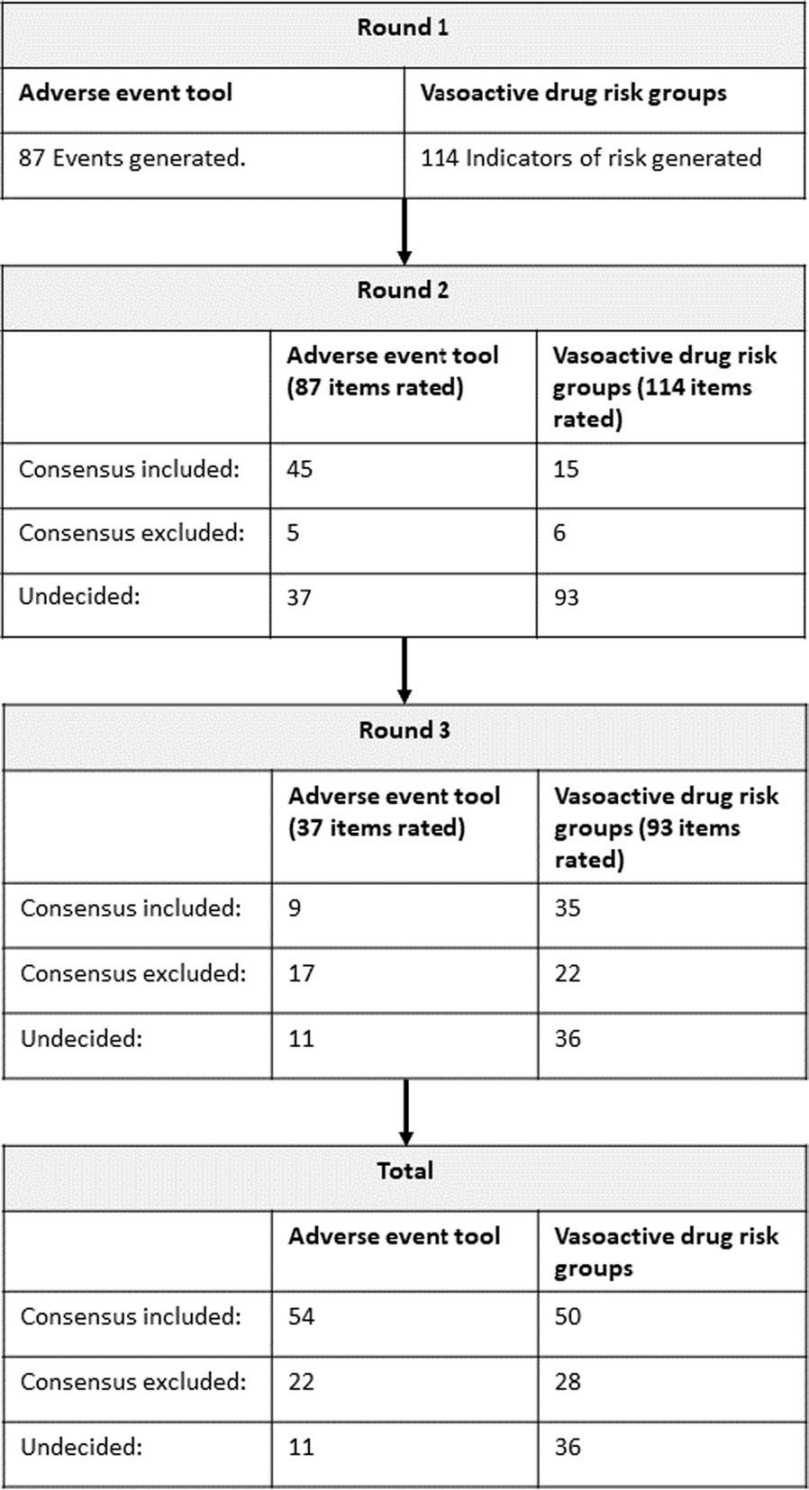
Participant responses to the three rounds of the Delphi process. 29 participants responded for rounds 1–3

Participant recruitment began in June 2017 and following this, round one questionnaires were sent out in October 2017, round two in December 2017 and round three in March 2018. The COVID-19 pandemic impacted upon timing of dissemination of results. After initial approach, 49 clinicians initially expressed interest in participation, of whom 25 were not enrolled and 24 agreed to participate. Ten ICU physicians, five nurses and nine physiotherapists/physical therapists were recruited, who were based in 10 different countries across five continents and had a mean 18 years of ICU experience (SD ± 8.1). Fourteen participants (61%) had published a median of 10 (IQR 3–17) peer-reviewed papers in the field of ICU rehabilitation (Table 1). Five former ICU patients and one patient relative were recruited from the UK to support the definition of an adverse event (Table 2). The participant response rate for all three rounds of the Delphi process was 97% with only one non-responder who was invited to respond to rounds one and two, but not round three. The responses to the three rounds are summarised in Fig. 2. The steering group decided to stop the Delphi process after the three rounds as the pre-specified end point of adequate consensus had been reached to form the adverse event and risk assessment tools.

**Table 1:** Clinician participant demographics

	Clinicians (N = 24)
Profession, n (%)	
Doctor	10 (42)
Nurse	5 (21)
Physiotherapist/physical therapist	9 (38)
Age (n = 22), mean (± SD)	45 (8.6)
Male (n = 21), n (%)	11 (52)
Country of work, n (%)	
Australia	7 (29)
Belgium	1 (4)
Canada	1 (4)
Germany	1 (4)
India	1 (4)
Japan	1 (4)
Netherlands	1 (4)
South Africa	1 (4)
UK	6 (25)
USA	4 (17)
Type of ICU (n = 23), n (%)	
General/mixed	21(91)
Specialist only	2 (9)
Number of years of ICU experience (n = 23), mean (± SD)	18 (8.1)
Number who specified academic position (n = 23), n (%)	8 (35)
Number who have published peer-reviewed papers in the field of ICU rehabilitation (n = 23), n (%)	14 (61)
Of published authors (n = 14), number of peer-reviewed papers published in the field of ICU rehabilitation, median (IQR)	10 (3–17)

*SD* standard deviation, *IQR* interquartile range

**Table 2:** Patient and relative participant demographics

	Former ICU patients and their relatives (N = 6)
Service user participants	
Patients, n (%)	5 (83)
Relatives, n (%)	1 (17)
Age, mean (± SD)	60 (8.7)
Male, n (%)	4 (67)
Patient ICU length of stay in days* (n = 5), mean (± SD)	37 (31.3)
Number of different hospitals experienced by patients (n = 5), n	6
Highest level of mobilisation experienced by patients (n = 5), n (%)	
None	1 (20)
Moving from bed to chair	1 (20)
Walking	3 (60)

*One participant reported their length of stay as approximate

*SD* standard deviation

### Adverse event definition

Round one resulted in 87 different suggestions of events to include in the adverse event tool after content analysis of responses was complete (see Additional File 1: Supplementary Table 1). An example illustrating how content analysis was carried out is contained in Additional File 1: Supplementary Table 2. These 87 events were rated by participants in round two, where 45 events reached consensus for inclusion and five consensus for exclusion. The steering group decided not to list five potential adverse events for patient/relative participants to rate, as they related to unsafe changes in physiological variables requiring clinical judgement of different values, therefore they were only judged by clinicians. The steering group decided for both Delphi questions, that any undecided items that directly contradicted other items that had reached consensus, would be excluded from the rest of the process. Four events were excluded after round two for this reason for the adverse event definition (see Additional File 1: Supplementary Table 3 for details). Only one event (‘large amount of chest secretions’) reached consensus where the majority patient/relative opinion conflicted with the whole group, with 72.4% of the whole group voting to exclude the event, but 66.7% of patients/relatives voting to include the event (Table 3). The steering group, including a former ICU patient, considered this event and decided not to return it to round three for re-rating. The remaining 37 un-decided events were re-rated in round three, where a further nine events reached consensus for inclusion. By the end of the process, 54 events reached consensus for inclusion, 22 for exclusion and 11 remained undecided (see Additional File 1: Supplementary Tables 3–5 for details of events and percentage consensus reached). One further event had reached consensus for exclusion (‘any respiratory deterioration) by 82.8% of the whole group; however, 66.7% of patients voted to include (Table 3). The steering group decided to keep to the majority group decision. Comments made by participants also underwent content analysis and the results are contained in the Additional File 1: Supplementary Table 6.

**Table 3:** Adverse events where majority patient ratings differ from the whole group

Adverse event	Participant ratings (%)		
	Strongly disagree + disagree	Undecided	Agree + strongly agree
Events that reached consensus after round 2			
Large amounts of chest secretions			
All participants	72.4	10.3	17.2
Clinicians	87.0	8.7	4.3
Patients	16.7	16.7	66.7
Events that reached consensus after round 3			
Any respiratory deterioration			
All participants	82.8	0	17.2
Clinicians	95.7	0	4.3
Patients	33.3	0	66.7
Undecided events after round 3 (with round 3 ratings)			
Any cardiovascular deterioration			
All participants	65.5	3.4	31.0
Clinicians	78.3	0	21.7
Patients	16.7	16.7	66.7
Dizziness due to cardiovascular deterioration			
All participants	68.9	24.1	6.8
Clinicians	78.2	17.4	4.3
Patients	33.3	50.0	16.7
Any unplanned movement of any indwelling devices, lines, tubes or drains			
All participants	51.7	10.3	37.9
Clinicians	60.9	8.7	30.4
Patients	16.7	16.7	66.7
Increased pain			
All participants	69.0	6.9	24.1
Clinicians	78.3	0	21.7
Patients	33.3	33.3	33.3
Agitation			
All participants	62.1	0	37.9
Clinicians	69.6	0	30.4
Patients	33.3	0	66.7
Patient distress			
All participants	51.7	6.9	41.3
Clinicians	65.2	4.3	30.4
Patients	0	16.7	83.3
Bearing weight inappropriately on an injured leg			
All participants	41.4	10.3	48.2
Clinicians	52.2	8.7	39.1
Patients	0	16.7	83.3
Increase in patient hallucinations			
All participants	58.6	3.4	37.9
Clinicians	69.6	4.3	26.1
Patients	16.7	0	83.3

All participants: n = 29. Clinicians: n = 23. Patients: n = 6

**Fig. 3:**
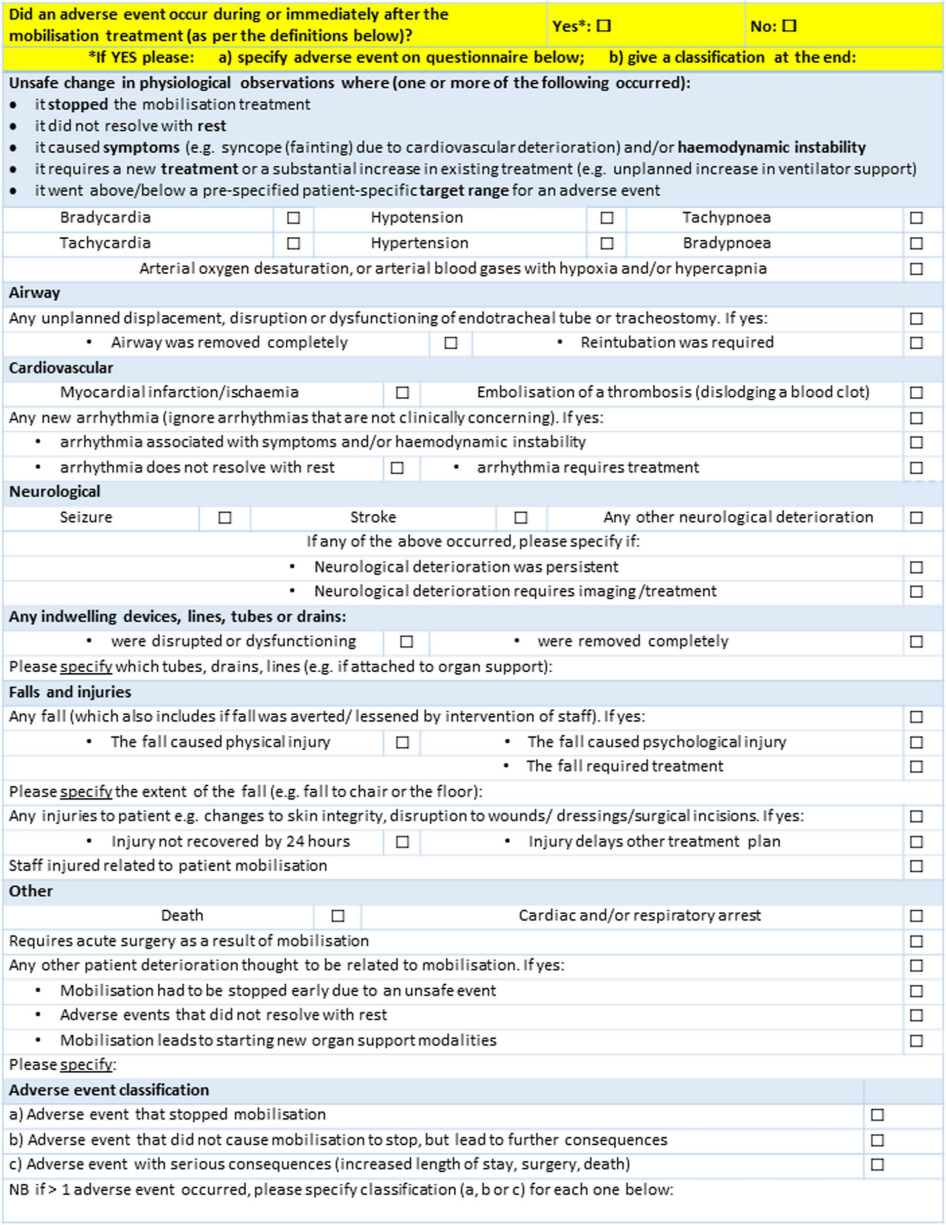
Final adverse event tool

Development of the adverse event tool included amalgamating the 54 events that reached consensus and wording changes in response to feedback; details of which can be found in Additional File 1. An example of how events were combined together is ‘changes to skin integrity’ given as an example of ‘any injuries to patient’ so in the tool it reads ‘Any injuries to patient e.g. changes to skin integrity…’. The final adverse event tool is presented in Fig. 3. The 54 events that reached consensus for inclusion fulfil study aim one as the clinician and patient agreement on the definition of an adverse event. The adverse event tool is a clear and concise representation of the adverse event definition.

### Risk assessment tool for physical rehabilitation with patient receiving vasoactive drugs

Round one generated 114 different indicators of risk (see Additional File 1: Supplementary Table 7). These related to general statements of how to assess risk and also specific risk indicators which were grouped as (a) those related to vasoactive drugs, (b) cardiovascular-specific indicators and (c) other indicators. The analysis process was challenging, particularly in relation to amalgamating the different suggestions for vasoactive drug dose. An example of how analysis was carried out is found in Additional File 1: Supplementary Table 8. The 114 indicators of risk were rated in round two, with 15 indicators of risk reaching consensus for inclusion and six for exclusion. This included one risk indicator excluded by the steering group despite not reaching consensus, as it contradicted an item that had reached consensus (see Additional File 1: Supplementary Table 9 for details). The remaining 93 undecided items were re-rated in round three, where a further 35 indicators of risk reached consensus for inclusion. Details of the 50 indicators of risk that reached consensus for inclusion in the final risk assessment tool by the end of the Delphi process, as well as the 28 that were excluded and the 36 left undecided are found in Additional File 1: Supplementary Tables 9–11. Eleven of the undecided indicators were then included because the sum of ratings for ‘agree’ plus ‘strongly agree’ (for higher risk) plus ‘contraindicated’ were ≥ 70% and they are found in Additional File 1: Supplementary Table 12. The results of content analysis of participant comments from rounds two and three are found in Additional File 1: Supplementary Table 13 for consideration alongside the final risk assessment tool.

**Fig. 4:**
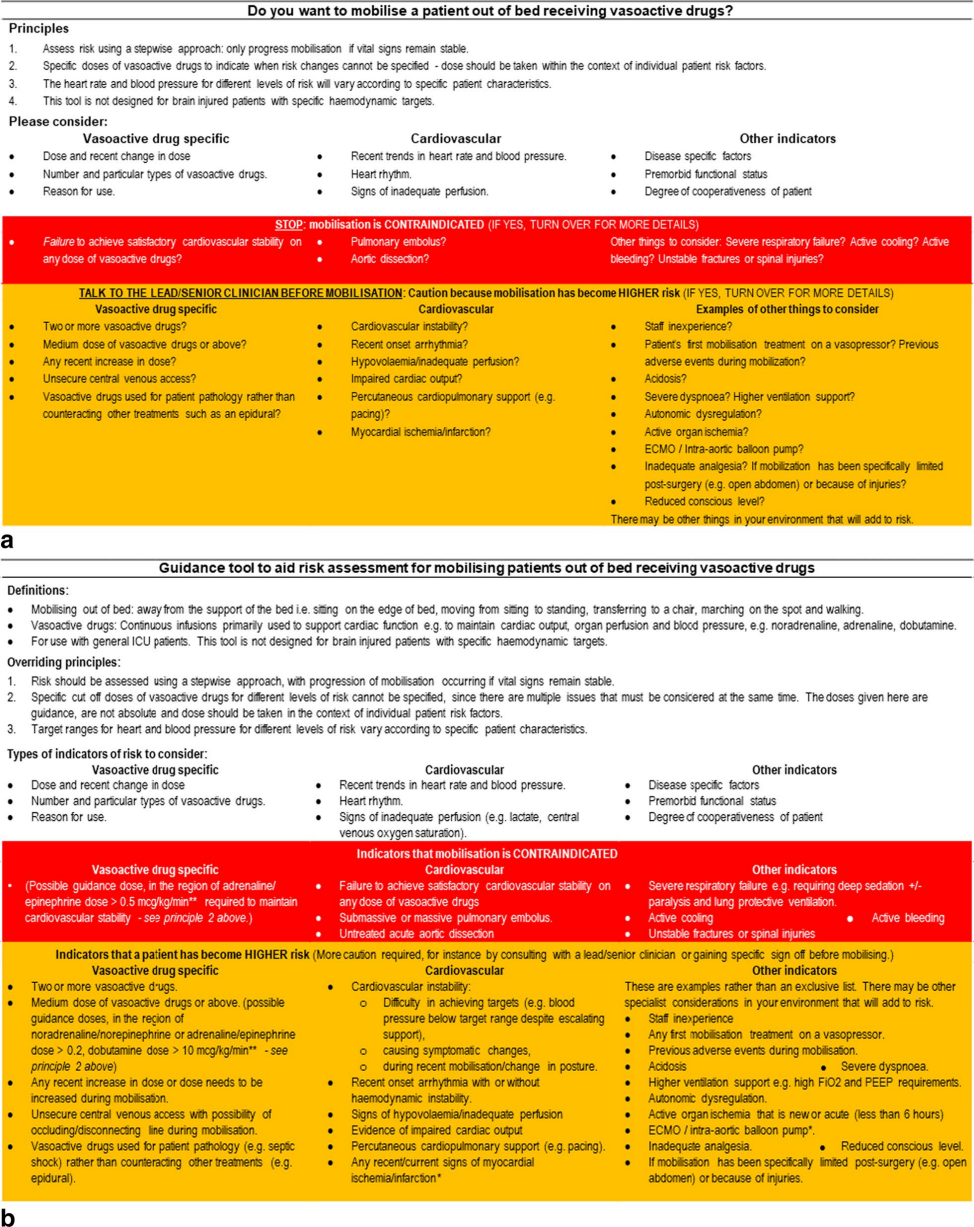
**a**: Final risk assessment tool initial summary page. Please note that this is a guidance tool only. When judging the risk of mobilising, the main priority is to evaluate the patient on an individual basis, with the assessment of the clinical team based on the context of specific patient circumstances outweighing the above principles. **b** *Intra-aortic balloon pump / ECMO / myocardial ischaemia/infarction have been voted in as higher risk, however it should be noted that they are specialist considerations for judging the risk of mobilising that are beyond the remit of this tool. In addition, in certain situations previous guidelines have judged them to be a contraindication [5]. **Drug dose assumes a typical weight of 70 kg, therefore please multiply by 70 to convert to mcg/min. Please note that this is a guidance tool therefore the contents are not absolutes or an exhaustive list. When judging the risk of mobilising, the main priority is to evaluate the patient on an individual basis, with the assessment of the clinical team based on the context of specific patient circumstances outweighing the above principles [1, 5]. **b**: Final risk assessment tool for rehabilitation for patients receiving vasoactive drugs

The risk assessment tool was developed from the 50 indicators of risks. One point of discussion was including the overarching principle that had reached consensus of not specifying firm cut off vasoactive drug doses for different levels of risk, when several dose thresholds also reached consensus for inclusion. The decision was made to emphasise the overarching principle and include the dose thresholds but to qualify them as signal doses that may guide individual risk judgement and should always be considered in the context of individual risk factors. Furthermore, some included indicators of risk were felt to be specialist considerations, such as for intra-aortic balloon pumps which has previously been considered a contraindication [5]. These were then highlighted as requiring more detailed assessment as they were beyond the remit of the tool. Finally, a ‘traffic-light’ formatting system [5] was utilised to enhance readability. Tool testing resulted in a few further minor wording and formatting changes. Examples of how items were combined and wording changes can be found in Additional File 1. To illustrate, ‘medium dose’ and ‘higher dose’ were combined to become ‘medium and above’ in the final tool. During testing, the usability of the tool was found to be compromised by its length; therefore, an initial summary page was created (see Additional File 1: Supplementary Fig. 1). The simplified summary is designed to be used alongside the main tool so that clinicians can link the results of the simplified summary back to the full risk assessment tool. The final tool is found in Fig. 4. The 50 indicators of risk that reached consensus for inclusion fulfil study aim two as Delphi panel consensus on defining risk characteristics for rehabilitation with ICU patients receiving vasoactive drugs. The risk assessment tool is a concise representation of these risk characteristics.

## Discussion

This study reached consensus within an international group of ICU clinicians and clinician researchers, as well as former ICU patients from the UK, on an adverse event tool to measure the safety of physical rehabilitation with patients on intensive care. Secondly, clinician and clinician researcher participants reached consensus on a risk assessment tool for rehabilitation away from the support of the bed for ICU patients receiving vasoactive drugs.

The adverse event tool moves away from using specific thresholds previously used to define unsafe changes in physiological variables [7, 8] and instead opts for individualised patient-specific target ranges or specific events such as causing rehabilitation to stop or requiring a new treatment. This avoids the problem of one threshold not being appropriate for all patient groups [60] and therefore, means the events captured are more meaningful. The majority patient opinion only disagreed with two events that reached consensus (which were excluded; however, the majority of patients voted to include). According to participant comments, these may have been excluded because ‘any respiratory deterioration’ lacked specificity and ‘large amount of chest secretions’ is not necessarily linked to an undesirable outcome of rehabilitation. Indeed, these have not been included in a previous adverse event tool [13] or in recent trials [4, 17]. This tool did not include more subjective adverse patient symptoms, previously noted in some studies, such as distress and agitation [11, 61, 62]. However, these should still be closely monitored by clinicians as patient-important, as they were suggested and agreed upon by patient participants [46]. Finally, this tool shares similarities with a previous tool produced by consensus conference [13]. However, this new tool builds on the previous tool, for example, by adding other physiological parameters (such as deranged heart rate and respiratory rate), myocardial infarction, neurological events, clarifying a less detailed falls classification and adding staff injuries. In addition, this tool includes and considers the opinion of service users.

Our study therefore provides an adverse event tool informed by clinicians and patients for use in future research. This is important as a lack of adverse event reporting has been found [63] and recent trials describing safety did not use a single way to define adverse events so results could be combined more robustly [4, 17, 64]. Furthermore, ongoing study of adverse events is important to clarify when safety is a concern as the largest ICU rehabilitation trial to date found an increase in adverse events when their intensive rehabilitation intervention was compared with usual care [4]. However, current meta-analysis has suggested that there is not a significant effect of rehabilitation on safety [63].

The risk assessment tool for rehabilitation with patients receiving vasoactive drugs builds on previous work by providing a detailed framework applicable to a specific sub-group of ICU patient, receiving vasoactive drug treatment. It agrees with previous work that emphases that rehabilitation is not contraindicated just by the presence of vasoactive drugs [1, 5, 65]; however, patient-specific ICU clinical team assessment of individual circumstances should outweigh indicators listed in the tool [1, 5]. This risk assessment tool contrasted with previous guidance [1, 54, 66] by not setting specific vasoactive drug dose or cardiovascular stability thresholds for risk assessment decision making. In regards to vasoactive drug dose, this study concurred with other guidance that gave principles such as medium doses and an increasing dose indicating higher risk [5, 29, 65, 67]. However, the medium dose as well as the possible guidance doses included in the tool are higher than previously used by other guidance [54, 68]. It should be noted that the tool indicates when there is increased risk, but it does not imply that doses below these thresholds are always free from risk for rehabilitation. In terms of cardiovascular stability, this tool builds on previous work by a detailed consideration of cardiovascular stability in relation to rehabilitation on vasoactive drugs [1, 54, 66]. It concurs with other guidance [5] by relating instability to patient-specific target ranges, symptoms and arrhythmias and builds upon this by adding instability during recent patient movement.

The adverse event tool can be used to promote consistent safety reporting in clinical studies of ICU rehabilitation, as well as local ICU rehabilitation implementation work. However, before this, the usability of the tool and the feasibility of implementing it in clinical studies requires testing [69]. Following this, it is important to measure reliability and validity [70, 71] to facilitate uptake in future work. Furthermore, it should be considered as a starting framework which can be added to [72] when applied to more specialist situations such as with patients receiving extracorporeal membrane oxygenation. The risk assessment tool can be used directly by ICU clinicians as a framework to guide decision making for when risk of rehabilitation whilst receiving vasoactive drugs is increased. However, it does not indicate when there is low risk, its risk-prediction is yet to be determined and therefore, the tool should not be seen as an exhaustive list of absolutes.

The strengths of this study include the range of participants involved, including the service user perspective for the adverse event tool and the excellent response rate achieved. Several potential limitations to this study should be considered. Firstly, differences in language interpretation between participants may have led to ambiguity in responses, although questionnaires were tested with clinicians with a knowledge of the international literature to minimise this. Furthermore, the participant group were self-selecting and service users were only recruited from the UK, which may have limited the perspectives gained [46]. However, these were pragmatic compromises to allow the study to be completed within resource constraints. It should be noted, that generalisability is impacted by the use of expert opinion using a small sample size of participants, which may not be representative of all international opinion. Furthermore, using an open first round limited the process to items participants suggested, even if they were unclear or unspecific, and also meant the process did not start with a review of the literature. However, clinician participants were expected to have expert knowledge and the open round enriched the process by allowing new suggestions to be made which may not have been considered before, for example by patient participants [21]. Finally, answering two research questions in one Delphi process led to a large volume of data. Content analysis was used to amalgamate items to make questionnaires concise to achieve the excellent response rate [52]. However, this meant that there was not capacity to define some items precisely, sometimes impacting the scope of the final tools. Additionally, we did not report changes in response for the large number of items rated across both rounds to keep reporting succinct and clear. The final tools were developed through testing with ICU clinicians from different professions; however, the adverse event tool was not tested with nurses.

## Conclusions

Using a robust consensus process with an excellent response rate from key stakeholders including international, multi-professional ICU clinicians and clinician researchers, and former ICU patients, agreement has been reached on the definition of an adverse event for measuring the safety of ICU rehabilitation. Secondly, agreement has been reached on what to assess for risk when undertaking rehabilitation away from the support of the bed for patients receiving vasoactive drugs. Tools were developed guided by this consensus, which now warrant further empirical testing to define acceptability as well as risk-precision.

### Supplementary Information

**Additional file 1:** Questionnaires and supplementary results  (PDF 1589 KB)

## Data Availability

All grouped data is provided in the supplementary tables. Individual responses and data cannot be made publicly available due to participant confidentiality requirements.

## Abbreviation

ICU: Intensive care unit
