# Supplementary material for: Assessing the safety of physical rehabilitation in critically ill patients: a Delphi study
Source: Crit Care. 2024 Apr 30;28:144. doi: 10.1186/s13054-024-04919-x (PMC11061934; doi:10.1186/s13054-024-04919-x)
Supplement: Supplementary file 1 — Additional file 1: Questionnaires and supplementary results (PDF 1589 KB) [file 13054_2024_4919_MOESM1_ESM.pdf]

Additional File 1

Methods supplementary materials 2
Round one questionnaire for clinicians 2
Round two questionnaire for clinicians 6
Round three questionnaire for clinicians 18
Example participant supporting information sheet 46
Results supplementary materials 47
Supplementary Table 1: Adverse event definition results of content analysis of round one 47
Supplementary Table 2: Adverse event definition example of round one content analysis process 49
Supplementary Table 3: Adverse events that reached consensus for exclusion from the adverse event definition 50
Supplementary Table 4: Adverse events that reached consensus for inclusion in the adverse event definition 51
Supplementary Table 5: Adverse events that did not reach consensus with a summary of round three responses 53
Supplementary Table 6: Results of content analysis of participant comments made in rounds two and three for the adverse event definition 54
Examples of changes made to adverse event tool in response to user feedback 61
Supplementary Table 7: Vasoactive drugs risk assessment tool results of content analysis of round one 62
Supplementary Table 8: Vasoactive drugs risk assessment tool example of round one content analysis process 65
Supplementary Table 9: Indicators of risk that reached consensus for exclusion from the risk assessment tool 66
Supplementary Table 10: Indicators of risk that reached consensus for inclusion in the risk assessment tool 67
Supplementary Table 11: Indicators of risk that did not reach consensus for the risk assessment tool, with a summary of round three responses 69
Supplementary Table 12: Undecided indicators of risk that were included as votes for ‘agree’ plus ‘strongly agree’ plus ‘contraindicated’ were greater than or equal to 70% 70
Supplementary Table 13: Results of content analysis of participant comments made in rounds two and three for the risk assessment tool 71
Examples of changes made to risk assessment tool in response to user feedback 76
Supplementary Figure 1: Risk assessment tool initial summary page 77

Methods supplementary materials

Round one questionnaire for clinicians

Study code**:**

For all ICU patients:

1. **a) What events do you consider important to include in a definition of an adverse event that may occur during mobilisation on an ICU?** Please provide a minimum of 5 events and a maximum of 20.

   -

   b) For the events you have listed above, what are the specific characteristics, changes, measures, thresholds etc. you would use to indicate an adverse event has happened?

   -

For ICU patients receiving vasoactive drugs

2. When judging whether to mobilise an ICU patient who is *currently receiving vasoactive drugs*, how do you decide whether they are:

a) At **low risk**: Expected to mobilise at low risk, you would be happy for these patients to be mobilised routinely, for instance without senior medical input.
b) At **higher risk**: More risk may be involved and you would expect the mobilising nurse/physiotherapist to be more cautious, for instance by consulting with a lead/senior clinician or gaining specific sign off before mobilising these patients.
c) **Contraindicated** for mobilisation: It is definitely too unsafe to mobilise as the risk is too high.

We have included some variables you may want to consider, however please do not feel constrained by them if you would not use them in your decision. Please write what *you* think is important. Where possible/appropriate, please be as specific as you can in your answers.

a) Judging patients at low risk when mobilising on vasoactive drugs

**Would you consider…**	**If so, what are your specific indicators of low risk?**
Vasoactive drug specifics:<br>• Particular doses?<br>• The amount of change in dose over time?<br>• Different drugs or combinations of vasoactive medication?<br>• The reason for giving the drug?<br>If so, please only specify for commonly used vasoactive drugs	
Haemodynamics:<br>• Signs of cardiovascular impairment/ instability?<br>If so, please write the key way you would measure if this was present in a patient and if there was a specific threshold value.	
Does anything else affect the risk of mobilising on vasoactive drugs?<br>• Other acute issues (e.g. presence of mechanical ventilation or renal replacement therapy)?<br>• Co-morbidities (e.g. cardiovascular co-morbidities)?	
Other factors?	

Further comments (optional):

Click here to enter text.

b) Judging patients at <u>higher risk</u> when mobilising on vasoactive drugs (consult lead/senior clinician)

**Would you consider…** (see previous table for examples)	**If so, what are your specific indicators of higher risk?**
Vasoactive drug specifics:<br><br>(If so, please only specify for commonly used vasoactive drugs)	
Haemodynamics:<br><br>(If so, please write the <u>key</u> way you would measure if this was present in a patient and if there was a specific threshold value.)	
Does anything else affect the risk of mobilising on vasoactive drugs?	
Other factors?	

<u>Further comments</u> (optional):

Click here to enter text.

c) Patients where mobilisation on vasoactive drugs is <u>contraindicated</u>

**Would you consider…**<br>(see table in part (a) for examples)	**If so, what are your specific indicators that mobilisation is contraindicated?**
Vasoactive drug specifics:<br><br>(If so, please only specify for commonly used vasoactive drugs)	
Haemodynamics:<br><br>(If so, please write the <u>key</u> way you would measure if this was present in a patient and if there was a specific threshold value.)	
Does anything else affect the risk of mobilising on vasoactive drugs?	
Other factors?	

<u>Further comments</u> (optional):

Click here to enter text.

Round two questionnaire for clinicians

Study code**:**

For all ICU patients, with mobilisation including exercises, bed mobility, sitting on the edge of the bed, moving from sitting to standing, marching on the spot, transferring to the chair and walking:

1. **What events do you consider important to include in a definition of an adverse event that may occur during mobilisation on an ICU?**

Please rate your agreement with whether each event listed below should be included in the adverse event definition.

Cardiovascular [heart/blood pressure] (please answer for all events)	Strongly Disagree 1	Disagree 2	Un-decided 3	Agree 4	Strongly Agree 5
Any cardiovascular deterioration [any heart and blood pressure deterioration]	☐	☐	☐	☐	☐
• Dizziness due to cardiovascular deterioration	☐	☐	☐	☐	☐
• Syncope [Fainting] due to cardiovascular deterioration	☐	☐	☐	☐	☐
• Increased need for vasoactive drug support [increased need for drugs that support heart and blood pressure]	☐	☐	☐	☐	☐
• Myocardial infarction/ischaemia [heart attack/angina]	☐	☐	☐	☐	☐

Arrhythmia [abnormal heart rhythm] (please answer for all events)	Strongly Disagree 1	Disagree 2	Un-decided 3	Agree 4	Strongly Agree 5
Any new arrhythmia [any new abnormal heart rhythm]	☐	☐	☐	☐	☐
• Ignore arrhythmias that are not clinically concerning	☐	☐	☐	☐	☐
• Include arrhythmias associated with symptoms and/or haemodynamic instability [only include abnormal heart rhythms associated with symptoms and/or abnormal blood pressure]	☒	☐	☐	☐	☐
• Include arrhythmias that do not resolve with rest	☐	☐	☐	☐	☐
• Include arrhythmias that require treatment	☐	☐	☐	☐	☐

Comments (optional):

Click here to enter text.

Respiratory [breathing]<br>(please answer for all events)	Strongly Disagree<br>1	Disagree<br>2	Un-decided<br>3	Agree<br>4	Strongly Agree<br>5
Any respiratory deterioration [any worsening breathing]	☐	☐	☐	☐	☐
• Extreme coughing [coughing fit]	☐	☐	☐	☐	☐
• Large amounts of chest secretions [phlegm]	☐	☐	☐	☐	☐
• Dyspnoea [difficulty with breathing/breathlessness]	☐	☐	☐	☐	☐
• Increased work of breathing [using increased energy to breathe]	☐	☐	☐	☐	☐
• Hypoxia and/or hypercapnia on arterial blood gases [abnormally low oxygen or high carbon dioxide in the body]	☐	☐	☐	☐	☐
• NIV-mask [breathing machine mask] is removed/dislodged	☐	☐	☐	☐	☐
• Accidental disconnection from mechanical ventilator [the breathing machine]	☐	☐	☐	☐	☐
• Ventilator dyssynchrony [differences in timing of breathing between patient and breathing machine]	☐	☐	☐	☐	☐
• Unplanned increase in ventilator support [unplanned increase in help needed from breathing machine]	☐	☐	☐	☐	☐

Comments (optional):

Click here to enter text.

Abnormal change in physiological parameters [values that measure how different body organs are working] (please answer for all events)	Strongly Disagree<br>1	Disagree<br>2	Un-decided<br>3	Agree<br>4	Strongly Agree<br>5
Bradycardia [heart rate becomes too low]	☐	☐	☐	☐	☐
Tachycardia [heart rate becomes too high]	☐	☐	☐	☐	☐
Hypotension [blood pressure becomes too low]	☐	☐	☐	☐	☐
Hypertension [blood pressure becomes too high]	☐	☐	☐	☐	☐
Arterial Oxygen Desaturation [amount of oxygen in the blood becomes too low]	☐	☐	☐	☐	☐
Bradypnoea [number of breaths per minute becomes too low]	☐	☐	☐	☐	☐
Tachypnoea [number of breaths per minute become too high]	☐	☐	☐	☐	☐
What counts as an unsafe change in physiological values? [How do you know if the above values are too low or too high?]					

Abnormal change in physiological parameters [values that measure how different body organs are working] (please answer for all events)	Strongly Disagree<br>1	Disagree<br>2	Un-decided<br>3	Agree<br>4	Strongly Agree<br>5
• If it stops the mobilisation treatment	☐	☐	☐	☐	☐
• If it does not resolve with rest	☐	☐	☐	☐	☐
• If it causes symptoms and/or haemodynamic instability [abnormal blood pressure]	☐	☐	☐	☐	☐
• If it requires treatment	☐	☐	☐	☐	☐
• If it goes above/below a specific threshold unsafe value e.g. heart rate > ‘x’ beats per minute	☐	☐	☐	☐	☐
• If it goes above/below a patient-specific target range	☐	☐	☐	☐	☐
• If it increases or decreases by a certain amount e.g. heart rate increases by ‘x beats per minute’ or ‘x%’	☐	☐	☐	☐	☐
• For tachycardia only: Define using age-predicted maximal heart rate?	☐	☐	☐	☐	☐

Comments (optional):

Click here to enter text.

Neurological [brain] (please answer for all events)	Strongly Disagree<br>1	Disagree<br>2	Un-decided<br>3	Agree<br>4	Strongly Agree<br>5
Any neurological deterioration [any worsening brain function]	☐	☐	☐	☐	☐
• Include if neurological deterioration is persistent [if it lasts for a long time]	☐	☐	☐	☐	☐
• Include if neurological deterioration requires imaging [requires a scan]/treatment.	☐	☐	☐	☐	☐
Seizure. [fit]	☐	☐	☐	☐	☐
Stroke	☐	☐	☐	☐	☐

Comments (optional):

Click here to enter text.

Airway [breathing tube] (please answer for all events)	Strongly Disagree<br>1	Disagree<br>2	Un-decided<br>3	Agree<br>4	Strongly Agree<br>5
Any unplanned displacement of endotracheal tube or tracheostomy from original position placed [any unplanned movement of the breathing tube (“airway”) from its original position]	☐	☐	☐	☐	☐
• Include the above if airway disrupted or dysfunctioning [stops working properly]	☐	☐	☐	☐	☐

• Include the above if airway removed completely	☐	☐	☐	☐	☐
• Include the above if reintubation required [if the breathing tube needs to be put back in]	☐	☐	☐	☐	☐

Comments (optional):

Click here to enter text.

Lines and attachments (please answer for all events)	Strongly Disagree 1	Disagree 2	Un-decided 3	Agree 4	Strongly Agree 5
Any unplanned movement of any indwelling devices, lines, tubes or drains.	☐	☐	☐	☐	☐
• Include the above if they are pulled, trapped or tangled	☐	☐	☐	☐	☐
• Include if they are disrupted or dysfunctioning [stop working properly]	☐	☐	☐	☐	☐
• Include if they are removed completely	☐	☐	☐	☐	☐
• Include only if they require replacement	☐	☐	☐	☐	☐
• Relevant tubes, drains, lines (e.g. if attached to organ support), cardiac devices should be specified separately on adverse event tool.	☐	☐	☐	☐	☐

Comments (optional):

Click here to enter text.

Falls (please answer for all events)	Strongly Disagree 1	Disagree 2	Un-decided 3	Agree 4	Strongly Agree 5
Any fall	☐	☐	☐	☐	☐
• Include if fall was averted/ minimised by assistance of staff	☐	☐	☐	☐	☐
• Include falls that cause physical injury	☐	☐	☐	☐	☐
• Include falls that cause psychological injury	☐	☐	☐	☐	☐
• Include falls requiring treatment	☐	☐	☐	☐	☐
• The extent of the fall is important to state (e.g. fall to chair or the floor)	☐	☐	☐	☐	☐

Injuries (please answer for all events)	Strongly Disagree 1	Disagree 2	Un-decided 3	Agree 4	Strongly Agree 5
Any injuries to patient	☐	☐	☐	☐	☐
• Include if injury not recovered by 24 hours	☐	☐	☐	☐	☐
• Include if injury delays other treatment plan	☐	☐	☐	☐	☐

Staff injuries related to patient mobilisation	☐	☐	☐	☐	☐

Comments (optional):

Click here to enter text.

Patient symptoms (please answer for all events)	Strongly Disagree 1	Disagree 2	Un-decided 3	Agree 4	Strongly Agree 5
Increased pain	☐	☐	☐	☐	☐
Anxiety	☐	☐	☐	☐	☐
Agitation	☐	☐	☐	☐	☐
Fatigue [tiredness]	☐	☐	☐	☐	☐
Patient distress (even if unable to fully communicate specific problem)	☐	☐	☐	☐	☐
Discomfort	☐	☐	☐	☐	☐
• Include all of the above patient symptoms only if they lead to a return to bed	☐	☐	☐	☐	☐

Comments (optional):

Click here to enter text.

Other events (please answer for all events)	Strongly Disagree 1	Disagree 2	Un-decided 3	Agree 4	Strongly Agree 5
Cardiac and/or respiratory arrest [the heart and/or lungs stop]	☐	☐	☐	☐	☐
Death	☐	☐	☐	☐	☐
Embolisation of a thrombosis [dislodging a blood clot]	☐	☐	☐	☐	☐
Any disruption to wounds / dressings / surgical incisions [cuts]	☐	☐	☐	☐	☐
Bearing weight inappropriately on an injured leg	☐	☐	☐	☐	☐
Requires acute surgery as a result of mobilisation.	☐	☐	☐	☐	☐
Changes to skin integrity [damage to the skin]	☐	☐	☐	☐	☐
Increase in patient's body temperature/fever	☐	☐	☐	☐	☐
A mobilising patient tries to leave the ward	☐	☐	☐	☐	☐
Patient refusal to cooperate	☐	☐	☐	☐	☐
Increase in patient hallucinations (e.g. to the extent they require restraint/sedation or they cause injury to themselves or the environment).	☐	☐	☐	☐	☐
A mobilising patient disrupts other patients or their care	☐	☐	☐	☐	☐

Viewing another patient mobilise causes a patient to do something inappropriate or increases their agitation (e.g. they try to adjust their treatment)	☐	☐	☐	☐	☐

Comments (optional):

Click here to enter text.

General statements (please answer for all)	Strongly Disagree<br>1	Disagree<br>2	Un-decided<br>3	Agree<br>4	Strongly Agree<br>5
Include adverse events if they do not resolve with rest.	☐	☐	☐	☐	☐
Mobilising results in a prolonged recovery time [e.g. takes longer to get their breath back]: more than twice the duration of the mobilising period	☐	☐	☐	☐	☐
Include if mobilisation has to be stopped early due to an unsafe event	☐	☐	☐	☐	☐
If mobilisation leads to an increase in any additional organ support modalities [if mobilisation leads to body organs needing medical treatment]	☐	☐	☐	☐	☐
The adverse event tool should state at the end: 'Include any other patient deterioration thought to be related to mobilisation'	☐	☐	☐	☐	☐
In the adverse event tool, should we put unsafe events into groups in the following ways?					
• Classify as a) events that stop mobilisation; b) events that lead to further consequences other than stopping; c) events with serious consequences (increased length of stay, surgery, death)	☐	☐	☐	☐	☐
• Classify as a) life-threatening event with serious risk of death; b) non-serious adverse event	☐	☐	☐	☐	☐

Comments (optional):

Click here to enter text.

<u>For ICU patients *receiving vasoactive drugs* and for *mobilisation away from the support of the bed*:</u>
(i.e. sitting on the edge of bed, moving from sitting to standing, transferring to a chair, marching on the spot and walking)

2. When judging whether to mobilise an ICU patient who is *currently receiving vasoactive drugs*, how do you decide whether they are:

a) At **low risk**: Expected to mobilise at low risk, therefore you would be happy for these patients to be mobilised routinely, for instance without senior medical input.
b) At **higher risk**: More risk may be involved and you would expect the mobilising nurse/physiotherapist/physical therapist to be more cautious, for instance by consulting with a lead/senior clinician or gaining specific sign off before mobilising these patients.
c) **Contraindicated** for mobilisation: It is definitely too unsafe to mobilise as the risk is too high.

Please exclude brain injured patients with specific haemodynamic targets from consideration.

Please rate your agreement with these **general statements**:

(Please answer for <u>all</u>)	Strongly Disagree 1	Disagree 2	Un-decided 3	Agree 4	Strongly Agree 5
What should be considered when judging the risk of mobilising patients receiving vasoactive drugs?					
• Vasoactive drug dose	☐	☐	☐	☐	☐
• Recent change in vasoactive drug dose	☐	☐	☐	☐	☐
• Number of vasoactive drugs	☐	☐	☐	☐	☐
• Particular types of vasoactive drugs	☐	☐	☐	☐	☐
• Reason for vasoactive drug use	☐	☐	☐	☐	☐
• Type of venous access (e.g. peripheral/route of central access)	☐	☐	☐	☐	☐
• Recent trends in heart rate and blood pressure	☐	☐	☐	☐	☐
• Heart rhythm	☐	☐	☐	☐	☐
• Signs of inadequate perfusion (e.g. lactate, central venous oxygen saturation)	☐	☐	☐	☐	☐
• Echo findings	☐	☐	☐	☐	☐
• Disease specific factors	☐	☐	☐	☐	☐
• Premorbid functional status	☐	☐	☐	☐	☐
• Unit culture and resources	☐	☐	☐	☐	☐
• Degree of cooperativeness of patient	☐	☐	☐	☐	☐

**General statements continued:** (Please answer for all)	Strongly Disagree 1	Disagree 2	Un-decided 3	Agree 4	Strongly Agree 5
For vasoactive drug dose:					
• Specific threshold doses should be specified for low, higher and contraindicated groups	☐	☐	☐	☐	☐
• Doses should just be considered as 'low', 'medium' or "high"	☐	☐	☐	☐	☐
• Unable to specify cut off doses for these different levels of risk, since there are multiple issues that must be considered at the same time.	☐	☐	☐	☐	☐
For heart rate and blood pressure:					
• Specify using specific thresholds for the low, higher risk and contraindicated groups.	☐	☐	☐	☐	☐
• Target ranges would vary according to specific patient characteristics	☐	☐	☐	☐	☐
Risk should be judged using a stepwise approach to mobilisation: progression to the next level occurring if vital signs remain stable.	☐	☐	☐	☐	☐
No patient is low risk mobilising on a vasoactive agent	☐	☐	☐	☐	☐
All out of bed mobilisation contraindicated for patients receiving vasoactive drugs	☐	☐	☐	☐	☐
No specific cut off thresholds between low and high risk, since there are multiple issues that must be considered at the same time.	☐	☐	☐	☐	☐

Comments (optional):

Click here to enter text.

Now please either:

- **Rate your agreement with whether the following factors indicate a patient has changed from low risk to higher risk (i.e. that a patient has become higher risk).**
- **Or, please specify if you feel the following factors indicate that mobilisation is contraindicated.**

Vasoactive drug specific indicators of risk: (Please answer for all)	Indicator of **higher risk?**					**Or** select if mobilising **contra-indicated**
	Strongly Disagree 1	Disagree 2	Un-decided 3	Agree 4	Strongly Agree 5	
Any *first* mobilisation treatment on a vasopressor	☐	☐	☐	☐	☐	☐
Previous adverse events during mobilization	☐	☐	☐	☐	☐	☐
Vasoactive drugs used for patient pathology rather than counteracting other treatments e.g. epidural	☐	☐	☐	☐	☐	☐
Unsecure central venous access or with possibility of occluding/disconnecting line during mobilisation	☐	☐	☒	☐	☐	☐

Vasoactive drug specific indicators of risk: (Please answer for all)	Indicator of **higher risk?** Strongly Disagree 1	Disagree 2	Un-decided 3	Agree 4	Strongly Agree 5	**Or** select if mobilising **contra-indicated**
Failure to achieve satisfactory cardiovascular stability on any dose of vasoactive drugs	☐	☐	☐	☐	☐	☐
**DOSES THAT ARE REQUIRED TO MAINTAIN CARDIOVASCULAR STABILITY**:						
Low dose vasoactive drugs	☐	☐	☐	☐	☐	☐
Medium dose of vasoactive drugs	☐	☐	☐	☐	☐	☐
Higher dose of vasoactive drugs	☐	☐	☐	☐	☐	☐
Low dose vasoactive drugs with a primary *inotropic* effect	☐	☐	☐	☐	☐	☐
Medium or high doses of vasoactive drugs with a primary *inotropic* effect	☐	☐	☐	☐	☐	☐
No recent increase in dose	☐	☐	☐	☐	☐	☐
Any recent increase in dose	☐	☐	☐	☐	☐	☐
Dose needs to be increased during mobilisation	☐	☐	☐	☐	☐	☐
Single vasoactive drug	☐	☐	☐	☐	☐	☐
Two vasoactive drugs	☐	☐	☐	☐	☐	☐
More than two vasoactive drugs	☐	☐	☐	☐	☐	☐
Noradrenaline/norepinephrine dose < 0.1 mcg/kg/min*	☐	☐	☐	☐	☐	☐
Noradrenaline/norepinephrine dose between 0.1-0.2 mcg/kg/min*	☐	☐	☐	☐	☐	☐
Noradrenaline/norepinephrine dose 0.2-0.5 mcg/kg/min*	☐	☐	☐	☐	☐	☐
Noradrenaline/norepinephrine dose > 0.5 mcg/kg/min*	☐	☐	☐	☐	☐	☐
Adrenaline/epinephrine dose < 0.1 mcg/kg/min*	☐	☐	☐	☐	☐	☐
Adrenaline/epinephrine dose between 0.1-0.2 mcg/kg/min*	☐	☐	☐	☐	☐	☐
Adrenaline/epinephrine dose 0.2-0.5 mcg/kg/min*	☐	☐	☐	☐	☐	☐
Adrenaline/epinephrine dose > 0.5 mcg/kg/min*	☐	☐	☐	☐	☐	☐
Dobutamine dose < 5 mcg/kg/min*	☐	☐	☐	☐	☐	☐
Dobutamine dose 5-10 mcg/kg/min*	☐	☐	☐	☐	☐	☐
Dobutamine dose > 10 mcg/kg/min*	☐	☐	☐	☐	☐	☐
Any dose adrenaline/epinephrine	☐	☐	☐	☐	☐	☐

Vasoactive drug specific indicators of risk: (Please answer for all)	Indicator of **higher risk?** Strongly Disagree 1	Disagree 2	Un-decided 3	Agree 4	Strongly Agree 5	**Or** select if mobilising **contra-indicated**
Any dose of dobutamine	☐	☐	☐	☐	☐	☐
Any dose of dopamine	☐	☐	☐	☐	☐	☐
Any dose of enoximone	☐	☐	☐	☐	☐	☐
Any dose of isoprenaline	☐	☐	☐	☐	☐	☐
Any dose of levosimendan	☐	☐	☐	☐	☐	☐
Any dose of noradrenaline/norepinephrine	☐	☐	☐	☐	☐	☐
Any dose of meteraminol	☐	☐	☐	☐	☐	☐
Any dose of milrinone	☐	☐	☐	☐	☐	☐
Any dose of vasopressin	☐	☐	☐	☐	☐	☐

**drug dose assumes a typical weight of 70kg, therefore please multiply by 70 to convert to mcg/min.*

Comments (optional):

Click here to enter text.

Cardiovascular indicators of risk (Please answer for all)	Indicator of **higher risk?** Strongly Disagree 1	Disagree 2	Un-decided 3	Agree 4	Strongly Agree 5	**Or** select if mobilising **contra-indicated**
Cardiovascular instability: difficulty in achieving targets	☐	☐	☐	☐	☐	☐
Cardiovascular instability causing symptomatic changes.	☐	☐	☐	☐	☐	☐
Cardiovascular instability during recent mobilisation/change in posture	☐	☐	☐	☐	☐	☐
Cardiovascular instability during any adjustment of vasoactive drug	☐	☐	☐	☐	☐	☐
Any Hypotension/Hypertension	☐	☐	☐	☐	☐	☐
Uncontrolled, symptomatic hypotension/ hypertension	☐	☐	☐	☐	☐	☐
Blood pressure below target range despite escalating support	☐	☐	☐	☐	☐	☐
Any tachycardia /bradycardia	☐	☐	☐	☐	☐	☐
New/recent onset arrhythmia with no haemodynamic compromise	☐	☐	☐	☐	☐	☐
New/recent onset of arrhythmias with hemodynamic instability	☐	☐	☐	☐	☐	☐
Arrhythmia that occurred >24 hours ago	☐	☐	☐	☐	☐	☐
Patient receiving anti-arrhythmic medication	☐	☐	☐	☐	☐	☐
Active requirement of fluid boluses	☐	☐	☐	☐	☐	☐

Signs of hypovolaemia/inadequate perfusion	☐	☐	☐	☐	☐	☐
Impaired cardiac output but pre-existing low cardiac output state	☐	☐	☐	☐	☐	☐
Evidence of impaired cardiac output	☐	☐	☐	☐	☐	☐
Any recent/current signs of myocardial ischemia/infarction	☐	☐	☐	☐	☐	☐
Increase in cardiothoracic ratio on chest x-ray film	☐	☐	☐	☐	☐	☐
Post-cardiac arrest	☐	☐	☐	☐	☐	☐
ECHO findings of submassive pulmonary embolus with right heart strain	☐	☐	☐	☐	☐	☐
Massive pulmonary emboli	☐	☐	☐	☐	☐	☐
Cardiovascular co-morbidities	☐	☐	☐	☐	☐	☐

Comments (optional):

Click here to enter text.

Other indicators of risk: (Please answer for all)	Indicator of **higher risk?**					**Or** select if mobilising **contra-indicated**
	Strongly Disagree 1	Disagree 2	Un-decided 3	Agree 4	Strongly Agree 5	
Patient in an acute phase of critical illness	☐	☐	☐	☐	☐	☐
Patient presents with an unclear diagnosis/ uncertain clinical course	☐	☐	☐	☐	☐	☐
Patients who are slowly improving from an episode of critical illness	☐	☐	☐	☐	☐	☐
Patient frailty	☐	☐	☐	☐	☐	☐
Patients receiving palliative care	☐	☐	☐	☐	☐	☐
First mobilisation post-surgery or after prolonged bed rest	☐	☐	☐	☐	☐	☐
If mobilization has been specifically limited post-surgery (e.g. open abdomen) or because of injuries	☐	☐	☐	☐	☐	☐
Inadequate analgesia	☐	☐	☐	☐	☐	☐
Autonomic dysregulation	☐	☐	☐	☐	☐	☐
Severe dyspnoea	☐	☐	☐	☐	☐	☐
Presence of an endotracheal tube	☐	☐	☐	☐	☐	☐
Requiring mechanical ventilation	☐	☐	☐	☐	☐	☐
Higher ventilation support required e.g. higher FiO2 and PEEP requirements	☐	☐	☐	☐	☐	☐

Other indicators of risk: (Please answer for all)	Indicator of **higher risk?** Strongly Disagree 1	Disagree 2	Un-decided 3	Agree 4	Strongly Agree 5	**Or** select if mobilising **contra-indicated**
Severe respiratory failure e.g. requiring deep sedation +/- paralysis and lung protective ventilation	☐	☐	☐	☐	☐	☐
Renal replacement therapy as long as not femoral access	☐	☐	☐	☐	☐	☐
Renal replacement therapy with femoral access	☐	☐	☐	☐	☐	☐
ECMO	☐	☐	☐	☐	☐	☐
Intra-aortic balloon pump	☐	☐	☐	☐	☐	☐
Percutaneous cardiopulmonary support (e.g. pacing)	☐	☐	☐	☐	☐	☐
Patient confusion/ delirium	☐	☐	☐	☐	☐	☐
Reduced conscious level	☐	☐	☐	☐	☐	☐
Obesity	☐	☐	☐	☐	☐	☐
Stroke	☐	☐	☐	☐	☐	☐
Recent episodes of pulmonary oedema	☐	☐	☐	☐	☐	☐
Recently active ischemia to limb (past 4-6 hours) now currently stable	☐	☐	☐	☐	☐	☐
Diagnosis of acute aortic dissection	☐	☐	☐	☐	☐	☐
pH acidotic on Blood Gas Analysis	☐	☐	☐	☐	☐	☐
Active organ ischemia that is new or acute (less than 6 hours)	☐	☐	☐	☐	☐	☐
Active cooling	☐	☐	☐	☐	☐	☐
Active bleeding	☐	☐	☐	☐	☐	☐
Unstable fractures or spinal injuries	☐	☐	☐	☐	☐	☐
Staff inexperience	☐	☐	☐	☐	☐	☐

Comments (optional):

Click here to enter text.

Round three questionnaire for clinicians

Due to the length of the round three questionnaires, they were split into two documents and participants were informed how long each took to complete to facilitate ease of use.

Questionnaire 3 – Part 1

Study code:

For all ICU patients, with mobilisation including exercises, bed mobility, sitting on the edge of the bed, moving from sitting to standing, marching on the spot, transferring to the chair and walking:

1. **What events do you consider important to include in a definition of an adverse event that may occur during mobilisation on an ICU?**

Please rate your agreement with whether each remaining event listed below should be included in the adverse event definition.

Cardiovascular [heart/blood pressure]

Events that have reached consensus for inclusion:

- **Syncope [Fainting] due to cardiovascular deterioration**
- **Myocardial infarction/ischaemia [heart attack/angina]**

Remaining events for rating:<br>(please answer for all events)	Strongly Disagree<br>1	Disagree<br>2	Un-decided<br>3	Agree<br>4	Strongly Agree<br>5
**Any cardiovascular deterioration [any heart and blood pressure deterioration]**					
Your previous rating:					
29 whole panel member's ratings from round 2:	0%	41.4%	3.4%	24.1%	31.0%
6 patient representative's ratings:	0%	0%	0%	16.7%	83.3%
23 clinicians' ratings:	0%	52.2%	4.3%	26.1%	17.4%
Comments: When defining a deterioration, consider that it is *abnormal* if heart rate and blood pressure does not change in response to exercise.					
Please re-rate your agreement here:	☐	☐	☐	☐	☐
• **Dizziness due to cardiovascular deterioration**					
Your previous rating:					
29 whole panel member's ratings from round 2 :	3.4%	24.1%	27.6%	24.1%	20.7%
6 patient representative's ratings:	0%	0%	16.7%	16.7%	66.7%
23 clinicians' ratings:	4.3%	30.4%	30.4%	26.1%	8.7%
Comments: Dizziness is not unusual, does not usually substantially impact upon the patient and settles if one takes things slowly.					
Please re-rate your agreement here:	☐	☐	☐	☐	☐
• **Increased need for vasoactive drug support [increased need for drugs that support heart and blood pressure]**					
Your previous rating:					
26 whole panel member's ratings from round 2:	0%	15.4%	19.2%	30.8%	34.6%
3 patient representative's ratings (3 unable to comment):	0%	0%	0%	66.7%	33.3%
23 clinicians' ratings:	0%	17.4%	21.7%	26.1%	34.8%
Comments:					

• An increase in vasoactive drug support can actually allow mobilisation to happen because it can be used to compensate for the usual decrease in blood pressure. So it is not necessarily an unsafe event that substantially impacts upon the patient.<br>• Only an unsafe event if in response to a heart/blood pressure deterioration or if increased above a certain amount.					
Please re-rate your agreement here:	☐	☐	☐	☐	☐

Arrhythmia [abnormal heart rhythm]

Events that have reached consensus for inclusion:

- **Include arrhythmias associated with symptoms and/or haemodynamic instability [only include abnormal heart rhythms associated with symptoms and/or abnormal blood pressure]**
- **Include arrhythmias that do not resolve with rest**
- **Include arrhythmias that require treatment**

Remaining events for rating:<br>(please answer for all events)	Strongly Disagree<br>1	Disagree<br>2	Un-decided<br>3	Agree<br>4	Strongly Agree<br>5
**Any new arrhythmia [any new abnormal heart rhythm]**					
Your previous rating:					
27 whole panel member's ratings from round 2:	0%	18.5%	18.5%	44.4%	18.5%
4 patient representative's ratings (2 unable to comment):	0%	0%	50.0%	0%	50.0%
23 clinicians' ratings:	0%	21.7%	13.0%	52.2%	13.0%
Please re-rate your agreement here:	☐	☐	☐	☐	☐
• **Ignore arrhythmias that are not clinically concerning**					
Your previous rating:					
27 whole panel member's ratings from round 2:	3.7%	11.1%	18.5%	55.6%	11.1%
4 patient representative's ratings (2 unable to comment):	0%	0%	25.0%	75.0%	0%
23 clinicians' ratings:	4.3%	13.0%	17.4%	52.2%	13.0%
Please re-rate your agreement here:	☐	☐	☐	☐	☐
Comments:<br>• Arrhythmias are not relevant as they are seldom related to mobilisation.<br>• It may be difficult for less experienced clinicians to decide what a clinically concerning arrhythmia is<br>• Premature ventricular contractions (PVC) [a certain type of abnormal heart rhythm] may not be clinically concerning and may or may not be related to immobility.<br>• We all have sinus tachycardia (ST) [a fast heart rate] when we exercise so that should not be considered an unsafe event in the ICU.<br>• Atrial fibrillation [a certain type of abnormal heart rhythm] is common in ICU patients and it is often debatable whether mobilisation caused it or if it was going to happen anyway.					

Comments (optional):

Click here to enter text.

Respiratory [breathing]

Events that have reached consensus for inclusion:

- **Hypoxia and/or hypercapnia on arterial blood gases [abnormally low oxygen or high carbon dioxide in the body]**
- **Unplanned increase in ventilator support [unplanned increase in help needed from breathing machine]**

Event that has reached consensus for exclusion:

- **Large amounts of chest secretions [phlegm]**

Remaining events for rating:<br>(please answer for all events)	Strongly Disagree<br>1	Disagree<br>2	Un-decided<br>3	Agree<br>4	Strongly Agree<br>5
**Any respiratory deterioration [any worsening breathing]**					
Your previous rating:					
28 whole panel member's ratings from round 2:	0%	46.4%	21.4%	21.4%	10.7%
5 patient representative's ratings (1 unable to comment):	0%	0%	0%	60.0%	40.0%
23 clinicians' ratings:	0%	56.5%	26.1%	13.0%	4.3%
Please re-rate your agreement here:	☐	☐	☐	☐	☐
• **Extreme coughing [coughing fit]**					
Your previous rating:					
29 whole panel member's ratings from round 2:	6.9%	37.9%	10.3%	41.4%	3.4%
6 patient representative's ratings:	0%	0%	0%	83.3%	16.7%
23 clinicians' ratings:	8.7%	47.8%	13.0%	30.4%	0%
Comments: It is not unusual for patients who mobilise to experience coughing as a result of opening up previously collapsed parts of the lung. It is a beneficial effect!					
Please re-rate your agreement here:	☐	☐	☐	☐	☐
• **Dyspnoea [difficulty with breathing/breathlessness]**					
Your previous rating:					
29 whole panel member's ratings from round 2:	0%	37.9%	13.8%	31.0%	17.2%
6 patient representative's ratings:	0%	0%	16.7%	33.3%	50.0%
23 clinicians' ratings:	0%	47.8%	13.0%	30.4%	8.7%
Comments: Breathlessness can be a normal response to exercise and not necessarily an unsafe event. It depends on the extent.					
Please re-rate your agreement here:	☐	☐	☐	☐	☐
• **Increased work of breathing [using increased energy to breathe]**					
Your previous rating:					
29 whole panel member's ratings from round 2:	3.4%	41.4%	20.7%	31.0%	3.4%
6 patient representative's ratings:	0%	16.7%	16.7%	66.7%	0%
23 clinicians' ratings:	4.3%	47.8%	21.7%	21.7%	4.3%

Remaining events for rating:<br>(please answer for all events)	Strongly Disagree<br>1	Disagree<br>2	Un-decided<br>3	Agree<br>4	Strongly Agree<br>5
Comments: Increased work of breathing can be a normal response to exercise and not necessarily an unsafe event. It depends on the extent.					
Please re-rate your agreement here:	☐	☐	☐	☐	☐
• **NIV-mask [breathing machine mask] is removed/dislodged**					
Your previous rating:					
29 whole panel member's ratings from round 2:	3.4%	51.7%	13.8%	20.7%	10.3%
6 patient representative's ratings:	0%	0%	50.0%	16.7%	33.3%
23 clinicians' ratings:	4.3%	65.2%	4.3%	21.7%	4.3%
Please re-rate your agreement here:	☐	☐	☐	☐	☐
• **Accidental disconnection from mechanical ventilator [the breathing machine]**					
Your previous rating:					
29 whole panel member's ratings from round 2:	3.4%	37.9%	13.8%	13.8%	31.0%
6 patient representative's ratings:	0%	0%	16.7%	0%	83.3%
23 clinicians' ratings:	4.3%	47.8%	13.0%	17.4%	17.4%
Comments: These events are rare and often trivial, e.g. if it is realised immediately and they are reconnected it may be fine. But it is potentially serious, so it is important to know whether it lead to anything bad actually happening to the patient.					
Please re-rate your agreement here:	☐	☐	☐	☐	☐
• **Ventilator dyssynchrony [differences in timing of breathing between patient and breathing machine]**					
Your previous rating:					
27 whole panel member's ratings from round 2:	0%	48.1%	22.2%	18.5%	11.1%
4 patient representative's ratings (2 unable to comment):	0%	0%	25.0%	25.0%	50.0%
23 clinicians' ratings:	0%	56.5%	21.7%	17.4%	4.3%
Comments: It is very uncommon for this to be significant in patients where the ventilator [breathing machine] has been adequately adjusted to the right settings. It depends on the extent.					
Please re-rate your agreement here:	☐	☐	☐	☐	☐
General comments:<br>• Some of these events may be very hard to define in order to measure as an unsafe event.<br>• Some of these events happen as a result of mobilisation but are not necessarily unsafe.<br>• Some are only unsafe if there is a clinical deterioration.<br>• Sometimes we want to push people a bit.					

Comments (optional):

Click here to enter text.

Abnormal change in physiological parameters [values that measure how different body organs are working]

Events that have reached consensus for inclusion:

- **Bradycardia [heart rate becomes too low]**
- **Tachycardia [heart rate becomes too high]**
- **Hypotension [blood pressure becomes too low]**
- **Hypertension [blood pressure becomes too high]**
- **Arterial Oxygen Desaturation [amount of oxygen in the blood becomes too low]**
- **Tachypnoea [number of breaths per minute become too high]**

What counts as an unsafe change in physiological values? [How do you know if the above values are too low or too high?]

- **If it stops the mobilisation treatment**
- **If it does not resolve with rest**
- **If it causes symptoms and/or haemodynamic instability [abnormal blood pressure]**
- **If it requires treatment**
- **If it goes above/below a patient-specific target range**

Remaining events for rating:<br>(please answer for all events)	Strongly Disagree<br>1	Disagree<br>2	Un-decided<br>3	Agree<br>4	Strongly Agree<br>5
**Bradypnoea [number of breaths per minute becomes too low]**					
Your previous rating:					
27 whole panel member's ratings from round 2:	0%	18.5%	18.5%	48.1%	14.8%
4 patient representative's ratings (2 unable to comment):	0%	0%	25.0%	50.0%	25.0%
23 clinicians' ratings:	0%	21.7%	17.4%	47.8%	13.0%
Comments: Why would a patient's breathing rate become too low during mobilisation? Bradypnoea must be incredibly uncommon.					
Please re-rate your agreement here:	☐	☐	☐	☐	☐
• **For tachycardia only: Define using age-predicted maximal heart rate?**					
Your previous rating:					
23 whole panel member's ratings from round 2:	4.3%	39.1%	26.1%	26.1%	4.3%
Patient representative's ratings:	Event not given to patients to rate.				
Comments: Age-predicted maximal heart rate is not a feasible approach for implementation for clinical practice or research.					
Please re-rate your agreement here:	☐	☐	☐	☐	☐
General comments:<br>• Changes in physiological values e.g. heart rate and blood pressure, can be a normal effect of exercise. This needs to be considered when deciding what is unsafe.<br>• Sometimes we want to push people a bit.					

Comments (optional):

Click here to enter text.

Neurological [brain]

Events that have reached consensus for inclusion:
- **Include if neurological deterioration is persistent [if it lasts for a long time]**
- **Include if neurological deterioration requires imaging [requires a scan]/treatment.**
- **Seizure. [fit]**
- **Stroke**

Remaining event for rating: (please answer for all events)	Strongly Disagree 1	Disagree 2	Un-decided 3	Agree 4	Strongly Agree 5
**Any neurological deterioration [any worsening brain function]**					
Your previous rating:					
29 whole panel member's ratings from round 2:	0%	10.3%	20.7%	41.4%	27.6%
6 patient representative's ratings:	0%	0%	0%	33.3%	66.7%
23 clinicians' ratings:	0%	13.0%	26.1%	43.5%	17.4%
Comments:<br>• I think it would be unlikely that the mobilisation would cause neurological events – it would happen anyway.<br>• As long as no other obvious reasons like low blood sugar can explain it.<br>• There are some neurological deteriorations that are less significant					
Please re-rate your agreement here:	☐	☐	☐	☐	☐

Comments (optional):

Click here to enter text.

Airway [breathing tube]

Events that have reached consensus for inclusion:
- **Any unplanned displacement of endotracheal tube or tracheostomy from original position placed [any unplanned movement of the breathing tube ("airway") from its original position]**
- **Include the above if airway disrupted or dysfunctioning [stops working properly]**
- **Include the above if airway removed completely**
- **Include the above if reintubation required [if the breathing tube needs to be put back in]**

Lines and attachments

Events that have reached consensus for inclusion:
- **Include if they are disrupted or dysfunctioning [stop working properly]**
- **Include if they are removed completely**
- **Relevant tubes, drains, lines (e.g. if attached to organ support), cardiac devices should be specified separately on adverse event tool.**

Remaining events for rating: (please answer for all events)	Strongly Disagree 1	Disagree 2	Un-decided 3	Agree 4	Strongly Agree 5
**Any unplanned movement of any indwelling devices, lines, tubes or drains.**					
Your previous rating:					
29 whole panel member's ratings from round 2:	0%	31.0%	20.7%	31.0%	17.2%
6 patient representative's ratings:	0%	0%	16.7%	33.3%	50.0%
23 clinicians' ratings:	0%	39.1%	21.7%	30.4%	8.7%

Please re-rate your agreement here:	☐	☐	☐	☐	☐
• **Include the above if they are pulled, trapped or tangled**					
Your previous rating:					
29 whole panel member's ratings from round 2:	3.4%	51.7%	20.7%	13.8%	10.3%
6 patient representative's ratings:	0%	0%	33.3%	33.3%	33.3%
23 clinicians' ratings:	4.3%	65.2%	17.4%	8.7%	4.3%
Comments: Tangling is less important than pulled or trapped. Tangled is more common with trips to the scanner than mobilisation.					
Please re-rate your agreement here:	☐	☐	☐	☐	☐
General comments: Not all attachments are of equal importance, for instance in terms of the risk to the patient if they are removed. Only important ones should be included.					

Comments (optional):

Click here to enter text.

Falls

Events that have reached consensus for inclusion:

- **Include falls that cause physical injury**
- **Include falls that cause psychological injury**
- **Include falls requiring treatment**
- **The extent of the fall is important to state (e.g. fall to chair or the floor)**

Remaining events for rating: (please answer for all events)	Strongly Disagree 1	Disagree 2	Un-decided 3	Agree 4	Strongly Agree 5
**Any fall**					
Your previous rating:					
29 whole panel member's ratings from round 2:	3.4%	24.1%	10.3%	27.6%	34.5%
6 patient representative's ratings:	0%	0%	16.7%	16.7%	66.7%
23 clinicians' ratings:	4.3%	30.4%	8.7%	30.4%	26.1%
Please re-rate your agreement here:	☐	☐	☐	☐	☐
• **Include if fall was averted/ minimised by assistance of staff**					
Your previous rating:					
29 whole panel member's ratings from round 2:	6.9%	31.0%	10.3%	48.3%	3.4%
6 patient representative's ratings:	0%	16.7%	16.7%	50.0%	16.7%
23 clinicians' ratings:	8.7%	34.8%	8.7%	47.8%	0%
Please re-rate your agreement here:	☐	☐	☐	☐	☐
Comments:<br>• We should simply measure whether there was a fall or not.<br>• Severity of fall and consequential damage should be noted.					

Injuries

Events that have reached consensus for inclusion:

- **Any injuries to patient**
- **Include if injury not recovered by 24 hours**
- **Include if injury delays other treatment plan**

Remaining events for rating: (please answer for all events)	Strongly Disagree 1	Disagree 2	Un-decided 3	Agree 4	Strongly Agree 5
**Staff injuries related to patient mobilisation**					
Your previous rating:					
29 whole panel member's ratings from round 2:	10.3%	13.8%	6.9%	20.7%	48.3%
6 patient representative's ratings:	0%	0%	33.3%	16.7%	50.0%
23 clinicians' ratings:	13.0%	17.4%	0%	21.7%	47.8%
Comments: Staff injuries are important but seem to be a quite different area that would require more detailed discussion to accurately measure.					
Please re-rate your agreement here:	☐	☐	☐	☐	☐

Comments (optional):

Click here to enter text.

Patient symptoms

Events for rating: (please answer for all events)	Strongly Disagree 1	Disagree 2	Un-decided 3	Agree 4	Strongly Agree 5
**Increased pain**					
Your previous rating:					
29 whole panel member's ratings from round 2:	0%	37.9%	24.1%	34.5%	3.4%
6 patient representative's ratings:	0%	0%	50.0%	50.0%	0%
23 clinicians' ratings:	0%	47.8%	17.4%	30.4%	4.3%
Comments: Pain and discomfort from trauma or operations need to be managed so that mobilisation can occur, but it is unrealistic to think that it will be absent.					
Please re-rate your agreement here:	☐	☐	☐	☐	☐
**Anxiety**					
Your previous rating:					
29 whole panel member's ratings from round 2:	0%	37.9%	31.0%	27.6%	3.4%
6 patient representative's ratings:	0%	0%	33.3%	50.0%	16.7%
23 clinicians' ratings:	0%	47.8%	30.4%	21.7%	0%
Please re-rate your agreement here:	☐	☐	☐	☐	☐
**Agitation**					
Your previous rating:					
29 whole panel member's ratings from round 2:	0%	34.5%	24.1%	41.4%	0%
6 patient representative's ratings:	0%	16.7%	33.3%	50.0%	0%

Events for rating: (please answer for all events)	Strongly Disagree 1	Disagree 2	Un-decided 3	Agree 4	Strongly Agree 5
23 clinicians' ratings:	0%	39.1%	21.7%	39.1%	0%
Comments: Agitation usually improves with mobilisation.					
Please re-rate your agreement here:	☐	☐	☐	☐	☐
**Fatigue [tiredness]**					
Your previous rating:					
28 whole panel member's ratings from round 2:	3.6%	57.1%	32.1%	7.1%	0%
6 patient representative's ratings:	0%	50.0%	16.7%	33.3%	0%
22 clinicians' ratings:	4.5%	59.1%	36.4%	0%	0%
Comments: Fatigue is a normal response to exercise and exercise usually helps with sleep. However one needs to be watchful for excessive fatigue due to inadequate rest between mobilisation sessions.					
Please re-rate your agreement here:	☐	☐	☐	☐	☐
**Patient distress (even if unable to fully communicate specific problem)**					
Your previous rating:					
29 whole panel member's ratings from round 2:	0%	34.5%	17.2%	34.5%	13.8%
6 patient representative's ratings:	0%	0%	16.7%	16.7%	66.7%
23 clinicians' ratings:	0%	43.5%	17.4%	39.1%	0%
Comments: Distress could be included even if it does not cause the patient to be returned to bed.					
Please re-rate your agreement here:	☐	☐	☐	☐	☐
**Discomfort**					
Your previous rating:					
28 whole panel member's ratings from round 2:	3.6%	50.0%	21.4%	21.4%	3.6%
6 patient representative's ratings:	0%	33.3%	33.3%	16.7%	16.7%
22 clinicians' ratings:	4.5%	54.5%	18.2%	22.7%	0%
Comments: Please see comment for 'increased pain'.					
Please re-rate your agreement here:	☐	☐	☐	☐	☐
• **Include all of the above patient symptoms only if they lead to a return to bed**					
Your previous rating:					
29 whole panel member's ratings from round 2:	3.4%	37.9%	20.7%	27.6%	10.3%
6 patient representative's ratings:	0%	16.7%	16.7%	33.3%	33.3%
23 clinicians' ratings:	4.3%	43.5%	21.7%	26.1%	4.3%
Please re-rate your agreement here:	☐	☐	☐	☐	☐

General comments:

- Many of these symptoms will be present to some degree in many patients and can usually be prevented if the clinician takes actions and educates patients prior to mobilization.
- Only an unsafe event if they interfere with treatment or are caused by another unsafe event
- These are weak definitions of unsafe events if they are just measured by clinician personal opinion of patient symptoms.

Comments (optional):

Click here to enter text.

Other events

Events that have reached consensus for inclusion:

- **Cardiac and/or respiratory arrest [the heart and/or lungs stop]**
- **Death**
- **Embolisation of a thrombosis [dislodging a blood clot]**
- **Requires acute surgery as a result of mobilisation.**
- **Changes to skin integrity [damage to the skin]**

Remaining events for rating: (please answer for all events)	Strongly Disagree 1	Disagree 2	Un-decided 3	Agree 4	Strongly Agree 5
**Any disruption to wounds / dressings / surgical incisions [cuts]**					
Your previous rating:					
29 whole panel member's ratings from round 2:	0%	20.7%	13.8%	37.9%	27.6%
6 patient representative's ratings:	0%	0%	16.7%	33.3%	50.0%
23 clinicians' ratings:	0%	26.1%	13.0%	39.1%	21.7%
Please re-rate your agreement here:	☐	☐	☐	☐	☐
**Bearing weight inappropriately on an injured leg**					
Your previous rating:					
29 whole panel member's ratings from round 2:	0%	24.1%	24.1%	20.7%	31.0%
6 patient representative's ratings:	0%	0%	16.7%	50.0%	33.3%
23 clinicians' ratings:	0%	30.4%	26.1%	13.0%	30.4%
Comments: Agree only if it results in a fracture or the need for other treatment.					
Please re-rate your agreement here:	☐	☐	☐	☐	☐
**Increase in patient's body temperature/fever**					
Your previous rating:					
28 whole panel member's ratings from round 2:	3.6%	60.7%	14.3%	14.3%	7.1%
5 patient representative's ratings (1 unable to comment):	0%	0%	20.0%	40.0%	40.0%
23 clinicians' ratings:	4.3%	73.9%	13.0%	8.7%	0%
Comments: Not sure this could ever be caused by mobilisation.					
Please re-rate your agreement here:	☐	☐	☐	☐	☐
**A mobilising patient tries to leave the ward**					
Your previous rating:					
28 whole panel member's ratings from round 2:	14.3%	42.9%	17.9%	17.9%	7.1%
6 patient representative's ratings:	0%	0%	33.3%	33.3%	33.3%
22 clinicians' ratings:	18.2%	54.5%	13.6%	13.6%	0%

Remaining events for rating: (please answer for all events)	Strongly Disagree 1	Disagree 2	Un-decided 3	Agree 4	Strongly Agree 5
Please re-rate your agreement here:	☐	☐	☐	☐	☐
**Patient refusal to cooperate**					
Your previous rating:					
29 whole panel member's ratings from round 2:	10.3%	55.2%	20.7%	10.3%	3.4%
6 patient representative's ratings:	0%	16.7%	50.0%	16.7%	16.7%
23 clinicians' ratings:	13.0%	65.2%	13.0%	8.7%	0%
Please re-rate your agreement here:	☐	☐	☐	☐	☐
**Increase in patient hallucinations (e.g. to the extent they require restraint/sedation or they cause injury to themselves or the environment).**					
Your previous rating:					
29 whole panel member's ratings from round 2:	0%	31.0%	20.7%	27.6%	20.7%
6 patient representative's ratings:	0%	0%	0%	16.7%	83.3%
23 clinicians' ratings:	0%	39.1%	26.1%	30.4%	4.3%
Comments: Not sure that mobilisation could cause this. This would be extremely unusual and usually there is a decrease in agitation with mobilisation. Hallucinations are difficult to evaluate in many non-verbal patients, so agitation may be a better term.					
Please re-rate your agreement here:	☐	☐	☐	☐	☐
**A mobilising patient disrupts other patients or their care**					
Your previous rating:					
29 whole panel member's ratings from round 2:	13.8%	41.4%	13.8%	20.7%	10.3%
6 patient representative's ratings:	0%	0%	16.7%	50.0%	33.3%
23 clinicians' ratings:	17.4%	52.2%	13.0%	13.0%	4.3%
Comments: This indicates inadequate supervision/health care rather than an adverse event of the mobilisation.					
Please re-rate your agreement here:	☐	☐	☐	☐	☐
**Viewing another patient mobilise causes a patient to do something inappropriate or increases their agitation (e.g. they try to adjust their treatment)**					
Your previous rating:					
28 whole panel member's ratings from round 2:	17.9%	46.4%	14.3%	14.3%	7.1%
5 patient representative's ratings (1 unable to comment):	0%	0%	0%	60.0%	40.0%
23 clinicians' ratings:	21.7%	56.5%	17.4%	4.3%	0%
Comments: This would be most likely in the context of inadequate nursing care/communication rather than an adverse event of the mobilisation.					
Please re-rate your agreement here:	☐	☐	☐	☐	☐

Comments (optional):

Click here to enter text.

General statements

Events that have reached consensus for inclusion:

- **Include if mobilisation has to be stopped early due to an unsafe event**
- **If mobilisation leads to an increase in any additional organ support modalities [if mobilisation leads to body organs needing medical treatment]**
- **The adverse event tool should state at the end: 'Include any other patient deterioration thought to be related to mobilisation'**
- **Classify as a) events that stop mobilisation; b) events that lead to further consequences other than stopping; c) events with serious consequences (increased length of stay, surgery, death)**

Remaining events for rating:<br>(please answer for all)	Strongly Disagree<br>1	Disagree<br>2	Un-decided<br>3	Agree<br>4	Strongly Agree<br>5
**Include adverse events if they do not resolve with rest.**					
Your previous rating:					
29 whole panel member's ratings from round 2:	3.4%	10.3%	20.7%	44.8%	20.7%
6 patient representative's ratings:	0%	0%	16.7%	50.0%	33.3%
23 clinicians' ratings:	4.3%	13.0%	21.7%	43.5%	17.4%
Comments: This would not make sense with some of the events listed above e.g. a stroke.					
Please re-rate your agreement here:	☐	☐	☐	☐	☐
**Mobilising results in a prolonged recovery time [e.g. takes longer to get their breath back]: more than twice the duration of the mobilising period**					
Your previous rating:					
29 whole panel member's ratings from round 2:	6.9%	41.4%	13.8%	31.0%	6.9%
6 patient representative's ratings:	0%	0%	16.7%	50.0%	33.3%
23 clinicians' ratings:	8.7%	52.2%	13.0%	26.1%	0%
Please re-rate your agreement here:	☐	☐	☐	☐	☐

Comments (optional):

Click here to enter text.

Questionnaire 3 – Part 2

Study code:

For ICU patients *receiving vasoactive drugs* and for *mobilisation away from the support of the bed*: (i.e. sitting on the edge of bed, moving from sitting to standing, transferring to a chair, marching on the spot and walking)

2. When judging whether to mobilise an ICU patient who is *currently receiving vasoactive drugs*, how do you decide whether they are:

a) At **low risk**: Expected to mobilise at low risk, therefore you would be happy for these patients to be mobilised routinely, for instance without senior medical input.
b) At **higher risk**: More risk may be involved and you would expect the mobilising nurse/physiotherapist/physical therapist to be more cautious, for instance by consulting with a lead/senior clinician or gaining specific sign off before mobilising these patients.
c) **Contraindicated** for mobilisation: It is definitely too unsafe to mobilise as the risk is too high.

Please exclude brain injured patients with specific haemodynamic targets from consideration.

Please rate your agreement with these remaining indicators of risk:

General statements

Indicators of risk that have reached consensus for inclusion:

What should be considered when judging the risk of mobilising patients receiving vasoactive drugs?

- **Vasoactive drug dose**
- **Recent change in vasoactive drug dose**
- **Number of vasoactive drugs**
- **Recent trends in heart rate and blood pressure**
- **Heart rhythm**
- **Signs of inadequate perfusion (e.g. lactate, central venous oxygen saturation)**
- **Disease specific factors**

Remaining indicators of risk for rating: (Please answer for all)	Strongly Disagree 1	Disagree 2	Un-decided 3	Agree 4	Strongly Agree 5
What should be considered when judging the risk of mobilising patients receiving vasoactive drugs?					
• **Particular types of vasoactive drugs**					
Your previous rating:					
23 panel member's ratings from round 2:	0%	26.1%	8.7%	34.8%	30.4%
Please re-rate your agreement here:	☐	☐	☐	☐	☐

Remaining indicators of risk for rating: (Please answer for all)	Strongly Disagree 1	Disagree 2	Un-decided 3	Agree 4	Strongly Agree 5
• **Reason for vasoactive drug use**					
Your previous rating:					
23 panel member's ratings from round 2:	0%	17.4%	13.0%	39.1%	30.4%
Please re-rate your agreement here:	☐	☐	☐	☐	☐
• **Type of venous access (e.g. peripheral/route of central access)**					
Your previous rating:					
23 panel member's ratings from round 2:	4.3%	52.2%	17.4%	17.4%	8.7%
Comments: I can't imagine a case with a patient on a vasoactive agent without central access.					
Please re-rate your agreement here:	☐	☐	☐	☐	☐
• **Echo findings**					
Your previous rating:					
23 panel member's ratings from round 2:	0%	30.4%	21.7%	39.1%	8.7%
Please re-rate your agreement here:	☐	☐	☐	☐	☐
• **Premorbid functional status**					
Your previous rating:					
23 panel member's ratings from round 2:	0%	21.7%	17.4%	52.2%	8.7%
Please re-rate your agreement here:	☐	☐	☐	☐	☐
• **Unit culture and resources**					
Your previous rating:					
23 panel member's ratings from round 2:	4.3%	39.1%	21.7%	30.4%	4.3%
Comments: Resources yes, unit culture no.					
Please re-rate your agreement here:	☐	☐	☐	☐	☐
• **Degree of cooperativeness of patient**					
Your previous rating:					
23 panel member's ratings from round 2:	4.3%	26.1%	8.7%	47.8%	13.0%
Please re-rate your agreement here:	☐	☐	☐	☐	☐
Comments: Combination with cardiac deterioration (especially cardiac output and arrhythmia) is important.					

General statements continued

Indicators of risk that have reached consensus for inclusion:

- **For heart rate and blood pressure: Target ranges would vary according to specific patient characteristics.**
- **Risk should be judged using a stepwise approach to mobilisation: progression to the next level occurring if vital signs remain stable.**

Indicator of risk that has reached consensus for exclusion:

- **All out of bed mobilisation contraindicated for patients receiving vasoactive drugs.**

Remaining indicators of risk for rating: (Please answer for all)	Strongly Disagree 1	Disagree 2	Un-decided 3	Agree 4	Strongly Agree 5
• **For vasoactive drug dose: Specific threshold doses should be specified for low, higher and contraindicated groups**					
Your previous rating:					
23 panel member's ratings from round 2:	13.0%	26.1%	13.0%	39.1%	8.7%
Please re-rate your agreement here:	☐	☐	☐	☐	☐
• **For vasoactive drug dose: Doses should just be considered as 'low', 'medium' or "high"**					
Your previous rating:					
23 panel member's ratings from round 2:	13.0%	30.4%	21.7%	30.4%	4.3%
Comments: I feel a classification of 'low', 'medium' or "high" is unclear.					
Please re-rate your agreement here:	☐	☐	☐	☐	☐
• **For vasoactive drug dose: Unable to specify cut off doses for these different levels of risk, since there are multiple issues that must be considered at the same time.**					
Your previous rating:					
23 panel member's ratings from round 2:	0%	21.7%	13.0%	39.1%	26.1%
Please re-rate your agreement here:	☐	☐	☐	☐	☐
**No patient is low risk mobilising on a vasoactive agent**					
Your previous rating:					
23 panel member's ratings from round 2:	13.0%	56.5%	8.7%	13.0%	8.7%
Please re-rate your agreement here:	☐	☐	☐	☐	☐
**No specific cut off thresholds between low and high risk, since there are multiple issues that must be considered at the same time.**					
Your previous rating:					
23 panel member's ratings from round 2:	8.7%	21.7%	34.8%	17.4%	17.4%
Please re-rate your agreement here:	☐	☐	☐	☐	☐
Comments: Often safer to mobilise on vasoactive agent than off it, as easier to titrate up to compensate for usual loss of orthostatic reflexes.					

Comments (optional):

Click here to enter text.

Now please either:

- **Rate your agreement with whether the following factors indicate a patient has changed from low risk to higher risk (i.e. that a patient has become higher risk).**
- **Or, please specify if you feel the following factors indicate that mobilisation is contraindicated.**

Vasoactive drug specific indicators of risk:

Factor that has reached consensus for inclusion as an indicator that a patient has changed from low risk to higher risk (i.e. that a patient has become higher risk):

- **Previous adverse events during mobilization**

Remaining indicators of risk for rating: (Please answer for all)	Indicator of **higher risk?** Strongly Disagree 1	Disagree 2	Un-decided 3	Agree 4	Strongly Agree 5	**Or** select if mobilising **contra-indicated**
**Any *first* mobilisation treatment on a vasopressor**						
Your previous rating:						
22 panel member's ratings from round 2:	13.6%	36.4%	9.1%	40.9%	0%	0%
Please re-rate your agreement here:	☐	☐	☐	☐	☐	☐
**Vasoactive drugs used for patient pathology rather than counteracting other treatments e.g. epidural**						
Your previous rating:						
23 panel member's ratings from round 2:	4.3%	21.7%	21.7%	43.5%	4.3%	4.3%
Please re-rate your agreement here:	☐	☐	☐	☐	☐	☐
**Unsecure central venous access or with possibility of occluding/disconnecting line during mobilisation**						
Your previous rating:						
23 panel member's ratings from round 2:	0%	13.0%	4.3%	47.8%	0%	34.8%
Comments: I would have thought secure central venous access was a standard of care. The available evidence suggests that theoretical concerns regarding kinking with mobilisation out of bed (e.g. femoral site) are unfounded.						
Please re-rate your agreement here:	☐	☐	☐	☐	☐	☐
**Failure to achieve satisfactory cardiovascular stability on any dose of vasoactive drugs**						
Your previous rating:						
23 panel member's ratings from round 2:	0%	0%	0%	21.7%	17.4%	60.9%
Please re-rate your agreement here:	☐	☐	☐	☐	☐	☐
DOSES THAT ARE REQUIRED TO MAINTAIN CARDIOVASCULAR STABILITY:						
**Low dose vasoactive drugs**						
Your previous rating:						
23 panel member's ratings from round 2:	4.3%	47.8%	30.4%	17.4%	0%	0%
Please re-rate your agreement here:	☐	☐	☐	☐	☐	☐

Remaining indicators of risk for rating: (Please answer for all)	Indicator of **higher risk?**					Or select if mobilising **contra-indicated**
	Strongly Disagree 1	Disagree 2	Un-decided 3	Agree 4	Strongly Agree 5	
**Medium dose of vasoactive drugs**						
Your previous rating:						
23 panel member's ratings from round 2:	4.3%	17.4%	26.1%	34.8%	13.0%	4.3%
Please re-rate your agreement here:	☐	☐	☐	☐	☐	☐
**Higher dose of vasoactive drugs**						
Your previous rating:						
23 panel member's ratings from round 2:	0%	8.7%	17.4%	30.4%	17.4%	26.1%
Please re-rate your agreement here:	☐	☐	☐	☐	☐	☐
**Low dose vasoactive drugs with a primary *inotropic* effect**						
Your previous rating:						
23 panel member's ratings from round 2:	4.3%	39.1%	34.8%	17.4%	4.3%	0%
Please re-rate your agreement here:	☐	☐	☐	☐	☐	☐
**Medium or high doses of vasoactive drugs with a primary *inotropic* effect**						
Your previous rating:						
23 panel member's ratings from round 2:	0%	21.7%	21.7%	30.4%	13.0%	13.0%
Please re-rate your agreement here:	☐	☐	☐	☐	☐	☐
**No recent increase in dose**						
Your previous rating:						
23 panel member's ratings from round 2:	13.0%	56.5%	4.3%	26.1%	0%	0%
Please re-rate your agreement here:	☐	☐	☐	☐	☐	☐
**Any recent increase in dose**						
Your previous rating:						
23 panel member's ratings from round 2:	0%	13.0%	26.1%	52.2%	4.3%	4.3%
Please re-rate your agreement here:	☐	☐	☐	☐	☐	☐
**Dose needs to be increased during mobilisation**						
Your previous rating:						
23 panel member's ratings from round 2:	4.3%	17.4%	17.4%	47.8%	8.7%	4.3%
Please re-rate your agreement here:	☐	☐	☐	☐	☐	☐
**Single vasoactive drug**						
Your previous rating:						
23 panel member's ratings from round 2:	13.0%	52.2%	17.4%	13.0%	4.3%	0%
Please re-rate your agreement here:	☐	☐	☐	☐	☐	☐
**Two vasoactive drugs**						
Your previous rating:						

Remaining indicators of risk for rating:<br>(Please answer for all)	Indicator of **higher risk?**<br>Strongly Disagree<br>1	Disagree<br><br>2	Un-decided<br>3	Agree<br><br>4	Strongly Agree<br>5	**Or select if** mobilising **contra-indicated**
23 panel member's ratings from round 2:	4.3%	17.4%	21.7%	34.8%	13.0%	8.7%
Please re-rate your agreement here:	☐	☐	☐	☐	☐	☐
**More than two vasoactive drugs**						
Your previous rating:						
23 panel member's ratings from round 2:	0%	17.4%	8.7%	34.8%	17.4%	21.7
Please re-rate your agreement here:	☐	☐	☐	☐	☐	☐
**Noradrenaline/norepinephrine dose < 0.1 mcg/kg/min***						
Your previous rating:						
23 panel member's ratings from round 2:	13.0%	43.5%	21.7%	17.4%	4.3%	0%
Please re-rate your agreement here:	☐	☐	☐	☐	☐	☐
**Noradrenaline/norepinephrine dose between 0.1-0.2 mcg/kg/min***						
Your previous rating:						
23 panel member's ratings from round 2:	0%	26.1%	39.1%	26.1%	4.3%	4.3%
Please re-rate your agreement here:	☐	☐	☐	☐	☐	☐
**Noradrenaline/norepinephrine dose 0.2-0.5 mcg/kg/min***						
Your previous rating:						
22 panel member's ratings from round 2:	0%	4.5%	27.3%	31.8%	9.1%	27.3%
Comments: Probably would not mobilise someone on >0.3 mcg/kg/min noradrenaline out of bed.						
Please re-rate your agreement here:	☐	☐	☐	☐	☐	☐
**Noradrenaline/norepinephrine dose > 0.5 mcg/kg/min***						
Your previous rating:						
23 panel member's ratings from round 2:	0%	0%	17.4%	17.4%	17.4%	47.8%
Please re-rate your agreement here:	☐	☐	☐	☐	☐	☐
**Adrenaline/epinephrine dose < 0.1 mcg/kg/min***						
Your previous rating:						
23 panel member's ratings from round 2:	8.7%	30.4%	30.4%	13.0%	8.7%	8.7%
Please re-rate your agreement here:	☐	☐	☐	☐	☐	☐
**Adrenaline/epinephrine dose between 0.1-0.2 mcg/kg/min***						
Your previous rating:						
23 panel member's ratings from round 2:	0%	21.7%	34.8%	21.7%	4.3%	17.4%
Please re-rate your agreement here:	☐	☐	☐	☐	☐	☐
**Adrenaline/epinephrine dose 0.2-0.5 mcg/kg/min***						
Your previous rating:						
22 panel member's ratings from round 2:	0%	4.5%	27.3%	13.6%	22.7%	31.8%

Remaining indicators of risk for rating:<br>(Please answer for all)	Indicator of **higher risk?**<br>Strongly Disagree<br>1	Disagree<br>2	Un-decided<br>3	Agree<br>4	Strongly Agree<br>5	**Or** select if mobilising **contra-indicated**
Comments: Probably would not mobilise someone on >0.3 mcg/kg/min adrenaline out of bed.						
Please re-rate your agreement here:	☐	☐	☐	☐	☐	☐
**Adrenaline/epinephrine dose > 0.5 mcg/kg/min***						
Your previous rating:						
23 panel member's ratings from round 2:	0%	0%	17.4%	17.4%	4.3%	60.9%
Please re-rate your agreement here:	☐	☐	☐	☐	☐	☐
**Dobutamine dose < 5 mcg/kg/min***						
Your previous rating:						
23 panel member's ratings from round 2:	4.3%	34.8%	26.1%	26.1%	8.7%	0%
Please re-rate your agreement here:	☐	☐	☐	☐	☐	☐
**Dobutamine dose 5-10 mcg/kg/min***						
Your previous rating:						
23 panel's member's ratings from round 2:	0%	17.4%	30.4%	26.1%	13.0%	13.0%
Please re-rate your agreement here:	☐	☐	☐	☐	☐	☐
**Dobutamine dose > 10 mcg/kg/min***						
Your previous rating:						
23 panel member's ratings from round 2:	0%	8.7%	26.1%	13.0%	17.4%	34.8%
Please re-rate your agreement here:	☐	☐	☐	☐	☐	☐
**Any dose adrenaline/epinephrine**						
Your previous rating:						
23 panel member's ratings from round 2:	17.4%	39.1%	17.4%	13.0%	4.3%	8.7%
Please re-rate your agreement here:	☐	☐	☐	☐	☐	☐
**Any dose of dobutamine**						
Your previous rating:						
23 panel member's ratings from round 2:	17.4%	39.1%	17.4%	21.7%	4.3%	0%
Please re-rate your agreement here:	☐	☐	☐	☐	☐	☐
**Any dose of dopamine**						
Your previous rating:						
23 panel member's ratings from round 2:	17.4%	47.8%	17.4%	13.0%	4.3%	0%
Please re-rate your agreement here:	☐	☐	☐	☐	☐	☐
**Any dose of enoximone**						
Your previous rating:						
23 panel member's ratings from round 2:	8.7%	30.4%	43.5%	8.7%	4.3%	4.3%
Please re-rate your agreement here:	☐	☐	☐	☐	☐	☐

Remaining indicators of risk for rating: (Please answer for all)	Indicator of **higher risk?**					**Or** select if mobilising **contra-indicated**
	Strongly Disagree 1	Disagree 2	Un-decided 3	Agree 4	Strongly Agree 5	
**Any dose of isoprenaline**						
Your previous rating:						
23 panel member's ratings from round 2:	13.0%	26.1%	39.1%	13.0%	4.3%	4.3%
Please re-rate your agreement here:	☐	☐	☐	☐	☐	☐
**Any dose of levosimendan**						
Your previous rating:						
23 panel member's ratings from round 2:	8.7%	34.8%	34.8%	13.0%	4.3%	4.3%
Comments: With levosimendan it depends on the indication.						
Please re-rate your agreement here:	☐	☐	☐	☐	☐	☐
**Any dose of noradrenaline/norepinephrine**						
Your previous rating:						
23 panel member's ratings from round 2:	17.4%	47.8%	13.0%	21.7%	0%	0%
Please re-rate your agreement here:	☐	☐	☐	☐	☐	☐
**Any dose of meteraminol**						
Your previous rating:						
23 panel member's ratings from round 2:	13.0%	34.8%	30.4%	17.4%	0%	4.3%
Please re-rate your agreement here:	☐	☐	☐	☐	☐	☐
**Any dose of milrinone**						
Your previous rating:						
23 panel member's ratings from round 2:	8.7%	30.4%	30.4%	21.7%	4.3%	4.3%
Comments: Tachycardia/tachyarrhythmia's can be problematic with milrinone (independent of any mobilisation), especially if one uses a loading dose.						
Please re-rate your agreement here:	☐	☐	☐	☐	☐	☐
**Any dose of vasopressin**						
Your previous rating:						
23 panel member's ratings from round 2:	8.7%	34.8%	17.4%	34.8%	4.3%	0%
Please re-rate your agreement here:	☐	☐	☐	☐	☐	☐
General comments: Answers based on patient severity for vasoactive drug selection.						

**drug dose assumes a typical weight of 70kg, therefore please multiply by 70 to convert to mcg/min.*

Comments (optional): Click here to enter text.

Cardiovascular indicators of risk

Factors that have reached consensus for inclusion as indicators that a patient has changed from low risk to higher risk (i.e. that a patient has become higher risk):

- **Cardiovascular instability causing symptomatic changes.**
- **Cardiovascular instability during recent mobilisation/change in posture**

Indicators of risk that have reached consensus for exclusion:

- **Arrhythmia that occurred >24 hours ago**
- **Patient receiving anti-arrhythmic medication**

Remaining indicators of risk for rating: (Please answer for all)	Indicator of **higher risk?**					**Or** select if mobilising **contra-indicated**
	Strongly Disagree 1	Disagree 2	Un-decided 3	Agree 4	Strongly Agree 5	
**Cardiovascular instability: difficulty in achieving targets**						
Your previous rating:						
23 panel member's ratings from round 2:	0%	0%	4.3%	43.5%	26.1%	26.1
Please re-rate your agreement here:	☐	☐	☐	☐	☐	☐
**Cardiovascular instability during any adjustment of vasoactive drug**						
Your previous rating:						
23 panel member's ratings from round 2:	0%	4.3%	8.7%	47.8%	21.7%	17.4
Comments: Depends on what the cardiovascular instability was.						
Please re-rate your agreement here:	☐	☐	☐	☐	☐	☐
**Any Hypotension/Hypertension**						
Your previous rating:						
23 panel member's ratings from round 2:	0%	47.8%	17.4%	34.8%	0%	0%
Please re-rate your agreement here:	☐	☐	☐	☐	☐	☐
**Uncontrolled, symptomatic hypotension/ hypertension**						
Your previous rating:						
23 panel member's ratings from round 2:	0%	4.3%	4.3%	34.8%	17.4%	39.1%
Please re-rate your agreement here:	☐	☐	☐	☐	☐	☐
**Blood pressure below target range despite escalating support**						
Your previous rating:						
22 panel member's ratings from round 2:	0%	4.5%	4.5%	31.8%	27.3%	31.8%
Please re-rate your agreement here:	☐	☐	☐	☐	☐	☐
**Any tachycardia /bradycardia**						
Your previous rating:						
23 panel member's ratings from round 2:	0%	43.5%	30.4%	21.7%	0%	4.3%
Please re-rate your agreement here:	☐	☐	☐	☐	☐	☐
**New/recent onset arrhythmia with no haemodynamic compromise**						
Your previous rating:						

Remaining indicators of risk for rating: (Please answer for all)	Indicator of **higher risk?**					**Or** select if mobilising **contra-indicated**
	Strongly Disagree 1	Disagree 2	Un-decided 3	Agree 4	Strongly Agree 5	
23 panel member's ratings from round 2:	8.7%	17.4%	13.0%	52.2%	4.3%	4.3%
Please re-rate your agreement here:	☐	☐	☐	☐	☐	☐
**New/recent onset of arrhythmias with hemodynamic instability**						
Your previous rating:						
23 panel member's ratings from round 2:	0%	0%	8.7%	34.8%	34.8%	21.7%
Please re-rate your agreement here:	☐	☐	☐	☐	☐	☐
**Active requirement of fluid boluses**						
Your previous rating:						
23 panel member's ratings from round 2:	0%	39.1%	13.0%	30.4%	13.0%	4.3%
Please re-rate your agreement here:	☐	☐	☐	☐	☐	☐
**Signs of hypovolaemia/inadequate perfusion**						
Your previous rating:						
23 panel member's ratings from round 2:	0%	17.4%	4.3%	39.1%	17.4%	21.7%
Please re-rate your agreement here:	☐	☐	☐	☐	☐	☐
**Impaired cardiac output but pre-existing low cardiac output state**						
Your previous rating:						
23 panel member's ratings from round 2:	0%	30.4%	21.7%	43.5%	0%	4.3%
Please re-rate your agreement here:	☐	☐	☐	☐	☐	☐
**Evidence of impaired cardiac output**						
Your previous rating:						
23 panel member's ratings from round 2:	0%	17.4%	8.7%	60.9%	0%	13.0%
Please re-rate your agreement here:	☐	☐	☐	☐	☐	☐
**Any recent/current signs of myocardial ischemia/infarction**						
Your previous rating:						
23 panel member's ratings from round 2:	0%	0%	8.7%	30.4%	26.1%	34.8%
Comments: Depends how recent the MI is.						
Please re-rate your agreement here:	☐	☐	☐	☐	☐	☐
**Increase in cardiothoracic ratio on chest x-ray film**						
Your previous rating:						
23 panel member's ratings from round 2:	8.7%	34.8%	34.8%	17.4%	4.3%	0%
Please re-rate your agreement here:	☐	☐	☐	☐	☐	☐
**Post-cardiac arrest**						
Your previous rating:						
23 panel member's ratings from round 2:	0%	26.1%	21.7%	21.7%	4.3%	26.1%

Remaining indicators of risk for rating: (Please answer for all)	Indicator of **higher risk?** Strongly Disagree 1	Disagree 2	Un-decided 3	Agree 4	Strongly Agree 5	**Or** select if mobilising **contra-indicated**
Please re-rate your agreement here:	☐	☐	☐	☐	☐	☐
**ECHO findings of submassive pulmonary embolus with right heart strain**						
Your previous rating:						
23 panel member's ratings from round 2:	0%	0%	8.7%	30.4%	17.4%	43.5%
Please re-rate your agreement here:	☐	☐	☐	☐	☐	☐
**Massive pulmonary emboli**						
Your previous rating:						
23 panel member's ratings from round 2:	0%	4.3%	0%	21.7%	21.7%	52.2%
Please re-rate your agreement here:	☐	☐	☐	☐	☐	☐
**Cardiovascular co-morbidities**						
Your previous rating:						
23 panel member's ratings from round 2:	8.7%	43.5%	17.4%	26.1%	4.3%	0%
Please re-rate your agreement here:	☐	☐	☐	☐	☐	☐
General comments: For some of these it depends on the situation or other factors not specified here.						

Comments (optional):
Click here to enter text.

Other indicators of risk:

Factors that have reached consensus for inclusion as indicators that a patient has changed from low risk to higher risk (i.e. that a patient has become higher risk):

- **Autonomic dysregulation**
- **Severe dyspnoea**
- **Higher ventilation support required e.g. higher FiO2 and PEEP requirements**

Indicators of risk that have reached consensus for exclusion:

- **Patients who are slowly improving from an episode of critical illness**
- **Obesity**

Remaining indicators of risk for rating: (Please answer for all)	Indicator of **higher risk?** Strongly Disagree 1	Disagree 2	Un-decided 3	Agree 4	Strongly Agree 5	**Or** select if mobilising **contra-indicated**
**Patient in an acute phase of critical illness**						
Your previous rating:						
23 panel member's ratings from round 2:	13.0%	26.1%	26.1%	26.1%	0%	8.7%
Please re-rate your agreement here:	☐	☐	☐	☐	☐	☐
**Patient presents with an unclear diagnosis/ uncertain clinical course**						
Your previous rating:						
23 panel member's ratings from round 2:	0%	43.5%	21.7%	21.7%	13.0%	0%
Please re-rate your agreement here:	☐	☐	☐	☐	☐	☐

Remaining indicators of risk for rating: (Please answer for all)	Indicator of **higher risk?**					**Or** select if mobilising **contra-indicated**
	Strongly Disagree 1	Disagree 2	Un-decided 3	Agree 4	Strongly Agree 5	
**Patient frailty**						
Your previous rating:						
23 panel member's ratings from round 2:	4.3%	65.2%	4.3%	26.1%	0%	0%
Please re-rate your agreement here:	☐	☐	☐	☐	☐	☐
**Patients receiving palliative care**						
Your previous rating:						
23 panel member's ratings from round 2:	4.3%	60.9%	26.1%	4.3%	0%	4.3
Please re-rate your agreement here:	☐	☐	☐	☐	☐	☐
**First mobilisation post-surgery or after prolonged bed rest**						
Your previous rating:						
23 panel member's ratings from round 2:	4.3%	60.9%	13.0%	21.7%	0%	0%
Please re-rate your agreement here:	☐	☐	☐	☐	☐	☐
**If mobilization has been specifically limited post-surgery (e.g. open abdomen) or because of injuries**						
Your previous rating:						
23 panel member's ratings from round 2:	4.3%	26.1%	8.7%	56.5%	0%	4.3%
Please re-rate your agreement here:	☐	☐	☐	☐	☐	☐
**Inadequate analgesia**						
Your previous rating:						
23 panel member's ratings from round 2:	4.3%	30.4%	4.3%	56.5%	0%	4.3%
Please re-rate your agreement here:	☐	☐	☐	☐	☐	☐
**Presence of an endotracheal tube**						
Your previous rating:						
23 panel member's ratings from round 2:	21.7%	34.8%	4.3%	21.7%	13.0%	4.3
Please re-rate your agreement here:	☐	☐	☐	☐	☐	☐
**Requiring mechanical ventilation**						
Your previous rating:						
23 panel member's ratings from round 2:	21.7%	39.1%	4.3%	26.1%	8.7%	0%
Please re-rate your agreement here:	☐	☐	☐	☐	☐	☐
**Severe respiratory failure e.g. requiring deep sedation +/- paralysis and lung protective ventilation**						
Your previous rating:						
23 panel member's ratings from round 2:	0%	8.7%	4.3%	13.0%	13.0%	60.9%
Please re-rate your agreement here:	☐	☐	☐	☐	☐	☐
**Renal replacement therapy as long as not femoral access**						

Remaining indicators of risk for rating: (Please answer for all)	Indicator of **higher risk?**					**Or** select if mobilising **contra-indicated**
	Strongly Disagree 1	Disagree 2	Un-decided 3	Agree 4	Strongly Agree 5	
Your previous rating:						
23 panel member's ratings from round 2:	13.0%	56.5%	13.0%	13.0%	4.3%	0%
Comments: The available evidence does not demonstrate any issues with renal replacement therapy and femoral access, and suggests improved filter life.						
Please re-rate your agreement here:	☐	☐	☐	☐	☐	☐
**Renal replacement therapy with femoral access**						
Your previous rating:						
23 panel member's ratings from round 2:	8.7%	26.1%	21.7%	17.4%	13.0%	13.0%
Comments: Please see comment above.						
Please re-rate your agreement here:	☐	☐	☐	☐	☐	☐
**ECMO**						
Your previous rating:						
23 panel member's ratings from round 2:	0%	21.7%	4.3%	30.4%	26.1%	17.4%
Please re-rate your agreement here:	☐	☐	☐	☐	☐	☐
**Intra-aortic balloon pump**						
Your previous rating:						
23 panel member's ratings from round 2:	0%	17.4%	0%	17.4%	13.0%	52.2%
Comments: Location affects feasibility and safety of some mobilization (femoral vs. axillary access).						
Please re-rate your agreement here:	☐	☐	☐	☐	☐	☐
**Percutaneous cardiopulmonary support (e.g. pacing)**						
Your previous rating:						
23 panel member's ratings from round 2:	4.3%	21.7%	0%	34.8%	17.4%	21.7%
Comments: Contraindicated if full dependence on temporary pacing wire.						
Please re-rate your agreement here:	☐	☐	☐	☐	☐	☐
**Patient confusion/ delirium**						
Your previous rating:						
23 panel member's ratings from round 2:	13.0%	30.4%	4.3%	43.5%	4.3%	4.3
Please re-rate your agreement here:	☐	☐	☐	☐	☐	☐
**Reduced conscious level**						
Your previous rating:						
23 panel member's ratings from round 2:	4.3%	17.4%	26.1%	30.4%	13.0%	8.7%
Comments: Drowsy patients can be mobilised out of bed which often improves drowsiness, but mobilising is more difficult if the patient is unconscious.						
Please re-rate your agreement here:	☐	☐	☐	☐	☐	☐
**Stroke**						

Remaining indicators of risk for rating: (Please answer for all)	Indicator of **higher risk?**					**Or** select if mobilising **contra-indicated**
	Strongly Disagree 1	Disagree 2	Un-decided 3	Agree 4	Strongly Agree 5	
Your previous rating:						
23 panel member's ratings from round 2:	13.0%	43.5%	8.7%	30.4%	4.3%	0%
Comments: The AVERT study suggests that intensive very early mobilisation may be associated with worse outcomes. We probably need a dose finding/timing/titration study.						
Please re-rate your agreement here:	☐	☐	☐	☐	☐	☐
**Recent episodes of pulmonary oedema**						
Your previous rating:						
23 panel member's ratings from round 2:	4.3%	39.1%	21.7%	30.4%	0%	4.3%
Comments: It depends on the nature of the episodes and likely underlying cause.						
Please re-rate your agreement here:	☐	☐	☐	☐	☐	☐
**Recently active ischemia to limb (past 4-6 hours) now currently stable**						
Your previous rating:						
23 panel member's ratings from round 2:	4.3%	34.8%	8.7%	34.8%	4.3%	13.0%
Please re-rate your agreement here:	☐	☐	☐	☐	☐	☐
**Diagnosis of acute aortic dissection**						
Your previous rating:						
23 panel member's ratings from round 2:	0%	8.7%	4.3%	21.7%	21.7%	43.5%
Please re-rate your agreement here:	☐	☐	☐	☐	☐	☐
**pH acidotic on Blood Gas Analysis**						
Your previous rating:						
23 panel member's ratings from round 2:	0%	26.1%	21.7%	34.8%	13.0%	4.3%
Please re-rate your agreement here:	☐	☐	☐	☐	☐	☐
**Active organ ischemia that is new or acute (less than 6 hours)**						
Your previous rating:						
23 panel member's ratings from round 2:	0%	8.7%	4.3%	39.1%	17.4%	30.4%
Please re-rate your agreement here:	☐	☐	☐	☐	☐	☐
**Active cooling**						
Your previous rating:						
23 panel member's ratings from round 2:	4.3%	4.3%	0%	26.1%	13.0%	52.2%
Please re-rate your agreement here:	☐	☐	☐	☐	☐	☐
**Active bleeding**						
Your previous rating:						
23 panel member's ratings from round 2:	0%	0%	0%	21.7%	17.4%	60.9%
Please re-rate your agreement here:	☐	☐	☐	☐	☐	☐
**Unstable fractures or spinal injuries**						

Remaining indicators of risk for rating: (Please answer for all)	Indicator of **higher risk?** Strongly Disagree 1	Disagree 2	Un-decided 3	Agree 4	Strongly Agree 5	**Or** select if mobilising **contra-indicated**
Your previous rating:						
23 panel member's ratings from round 2:	0%	4.3%	0%	8.7%	21.7%	65.2%
Please re-rate your agreement here:	☐	☐	☐	☐	☐	☐
**Staff inexperience**						
Your previous rating:						
23 panel member's ratings from round 2:	4.3%	21.7%	0%	43.5%	21.7%	8.7%
Comments: This is a staff factor and is not suitable for a patient's risk judgement.						
Please re-rate your agreement here:	☐	☐	☐	☐	☐	☐
General comments: Whilst many increase risk, it would not prevent mobilisation, just requires more planning.						

Comments (optional):

Click here to enter text.

Example participant supporting information sheet

Questionnaire supporting information - clinicians

1. Defining an adverse event that may occur <u>during</u> mobilisation on ICU

This definition should be feasible to use as an outcome in future trials and for clinicians to use to measure safety of their mobilisation treatments. For this definition, mobilisation is defined as including exercises, bed mobility, sitting on the edge of the bed, moving from sitting to standing, marching on the spot, transferring to the chair and walking. To give an idea, here are some examples of adverse events, although you do not have to use these when filling out the questionnaire; you can say *anything* which you think is important.

- General events: Events such as a fall, death, extubation, removal of lines and attachments or a cardiorespiratory arrest.
- Physiological events: Events such as unsafe changes in blood pressure, heart rate, oxygen saturation or respiratory rate.
- Other medical events may include a new arrhythmia, myocardial infarction or mechanical ventilation concerns.

2. Judging whether to mobilise an ICU patient who is *currently receiving vasoactive drugs.*

Please consider for a general ICU population and exclude brain injured patients with specific haemodynamic targets from consideration. For this question mobilisation is defined as mobilisation away from the support of the bed i.e. sitting on the edge of bed, moving from sitting to standing, transferring to a chair, marching on the spot and walking. The concept of the three ICU patient ‘risk groups’ is similar to that used in Hodgson et al. (Critical Care 2014; 18:658), however there are differences in the definitions of the groups used in this study.

Results supplementary materials

Supplementary Table 1: Adverse event definition results of content analysis of round one

Round 1 results			
**Unsafe change in physiological observations**			
Bradycardia<br>Tachycardia	Hypotension<br>Hypertension	Bradypnoea<br>Tachypnoea	Arterial Oxygen<br>Desaturation
What counts as an unsafe change in physiological values?<br>• If it stops the mobilisation treatment<br>• If it does not resolve with rest<br>• If it causes symptoms and/or haemodynamic instability<br>• If it requires treatment<br>• If it goes above/below a specific threshold unsafe value e.g. heart rate > ‘x’ beats per minute.<br>• If it goes above/below a patient-specific target range<br>• If it increases or decreases by a certain amount e.g. heart rate increases by ‘x beats per minute’ or ‘x%’<br>• For tachycardia only: Define using age-predicted maximal heart rate?			
**Airway**			
Any unplanned displacement of endotracheal tube or tracheostomy from original position placed<br>• Include the above if airway disrupted or dysfunctioning<br>• Include the above if airway removed completely<br>• Include the above if reintubation required			
**Cardiovascular**			
Any cardiovascular deterioration<br>• Dizziness due to cardiovascular deterioration<br>• Syncope due to cardiovascular deterioration<br>• Increased need for vasoactive drug support<br>• Myocardial infarction/ischaemia			
**Arrhythmia**			
Any new arrhythmia<br>• Ignore arrhythmias that are not clinically concerning<br>• Include arrhythmias associated with symptoms and/or haemodynamic instability<br>• Include arrhythmias that do not resolve with rest<br>• Include arrhythmias that require treatment			
**Respiratory**			
Any respiratory deterioration<br>• Extreme coughing<br>• Large amounts of chest secretions<br>• Dyspnoea<br>• Increased work of breathing<br>• Hypoxia and/or hypercapnia on arterial blood gases<br>• NIV-mask is removed/dislodged<br>• Accidental disconnection from mechanical ventilator<br>• Ventilator dyssynchrony<br>• Unplanned increase in ventilator support			
**Neurological**			
Any neurological deterioration<br>• Include if neurological deterioration is persistent<br>• Include if neurological deterioration requires imaging			

Seizure	Stroke

Lines and attachments

Any unplanned movement of any indwelling devices, lines, tubes or drains.

- Include the above if they are pulled, trapped or tangled
- Include if they are disrupted or dysfunctioning
- Include if they are removed completely
- Include only if they require replacement
- Relevant tubes, drains, lines (e.g. if attached to organ support), cardiac devices should be specified separately on adverse event tool.

Falls

Any fall

- Include if fall was averted/ minimised by assistance of staff
- Include falls that cause physical injury
- Include falls that cause psychological injury
- Include falls requiring treatment
- The extent of the fall is important to state (e.g. fall to chair or the floor)

Injuries

Any injuries to patient

- Include if injury not recovered by 24 hours
- Include if injury delays other treatment plan

Staff injuries related to patient mobilisation

Patient symptoms

Increased pain	Fatigue	Agitation
Anxiety	Discomfort	

Patient distress (even if unable to fully communicate specific problem)

- Include all of the above patient symptoms only if they lead to a return to bed

Other events

Cardiac and/or respiratory arrest	Death
Changes to skin integrity	Embolisation of a thrombosis

Any disruption to wounds / dressings / surgical incisions
Bearing weight inappropriately on an injured leg
Requires acute surgery as a result of mobilisation
Increase in patient's body temperature/fever
A mobilising patient tries to leave the ward
Patient refusal to cooperate
Increase in patient hallucinations (e.g. to the extent they require restraint/sedation or they cause injury to themselves or the environment)
A mobilising patient disrupts other patients or their care
Viewing another patient mobilise causes a patient to do something inappropriate or increases their agitation (e.g. they try to adjust their treatment)

General statements

Include adverse events if they do not resolve with rest.
Mobilising results in a prolonged recovery time: more than twice the duration of the mobilising period
Include if mobilisation has to be stopped early due to an unsafe event
If mobilisation leads to an increase in any additional organ support modalities
The adverse event tool should state at the end: 'Include any other patient deterioration thought to be related to mobilisation'
In the adverse event tool, should we put unsafe events into groups in the following ways?

- Classify as a) events that stop mobilisation; b) events that lead to further consequences other than stopping; c) events with serious consequences (increased length of stay, surgery, death)
- Classify as a) life-threatening event with serious risk of death; b) non-serious adverse event

Supplementary Table 2: Adverse event definition example of round one content analysis process

Original data	Interim categories	Wider category classifications / final events for rating
Original data related to tachycardia<br>• Heart rate more than 120 beats/min.<br>• pulse raised above 120 or by 50%<br>• 5 minutes after cessation of mobilisation<br>• HR >150/min<br>• Tachycardia<br>• High Heart frequency<br>• Heart frequency: > 220 - age<br>• Heart rate >130 beats per minute x 5 minutes<br>• increase in heart rate<br>• Significant increase in HR<br>• tachycardia<br>• Exceeding 80% of patients max predicted HR<br>• HR > 130<br>• unacceptable changes in heart rate… changes of more than 20 beats per minute in heart rate<br>• Increase HR >20% baseline if >100bpm<br>• Heart Rate Abnormality HR greater than or equal to 130 beats / min<br>• Abnormal response to activity that does not resolve with rest.<br>○ HR > 70% APMHR<br>○ >130 beats/minute<br>• Symptomatic and significant changes in heart rate or blood pressure causing hemodynamic instability requiring immediate medical intervention<br>• Symptomatic changes in heart rate and blood pressure without causing significant decompensation or hemodynamic instability not requiring immediate medical intervention	• Tachycardia<br>• -Symptomatic tachycardia<br>• -significant tachycardia<br>• -causing hemodynamic instability<br>• -requiring immediate medical intervention<br>• -without causing significant decompensation<br>• -without causing hemodynamic instability<br>• -not requiring immediate medical intervention<br>• -that does not resolve with rest<br>• -for 5 minutes duration<br>• -5 minutes after cessation of mobilisation<br>• -Heart rate more than 120 beats/min.<br>• -Heart rate >130 beats per minute<br>• -HR >150 beats/min<br>• -Heart rate raised by 50%<br>• -increase of more than 20 beats per minute in heart rate<br>• -Heart rate > 220 - age<br>• -Exceeding 80% of patients max predicted HR<br>• -Increase HR >20% baseline if >100bpm<br>• -HR > 70% age-predicted maximal heart rate	Tachycardia<br>What counts as an unsafe change?<br>• If it stops the mobilisation treatment<br>• If it does not resolve with rest<br>• If it causes symptoms and/or haemodynamic instability<br>• If it requires treatment<br>• If it goes above/below a specific threshold unsafe value e.g. heart rate > 'x' beats per minute.<br>• If it goes above/below a patient-specific target range<br>• If it increases or decreases by a certain amount e.g. heart rate increases by 'x beats per minute' or 'x%'<br>• Define using age-predicted maximal heart rate?

Supplementary Table 3: Adverse events that reached consensus for exclusion from the adverse event definition

Excluded adverse events	Percentage consensus
**Unsafe change in physiological observations**	
What counts as an unsafe change in physiological values?	
• If it goes above/below a specific threshold unsafe value e.g. heart rate > 'x' beats per minute	17.4%*
• If it increases or decreases by a certain amount e.g. heart rate increases by 'x beats per minute' or 'x%'	26.1%*
**Respiratory**	
Any respiratory deterioration	82.8%
Extreme coughing	75.8%
Large amounts of chest secretions	72.4%
Dyspnoea	75.9%
Increased work of breathing	82.7%
NIV-mask is removed/dislodged	93.1%
Accidental disconnection from mechanical ventilator	86.2%
Ventilator dyssynchrony	85.7%
**Lines and attachments**	
[Lines and attachments] Include if they are pulled, trapped or tangled	79.3%
[Lines and attachments] Include only if they require replacement	34.4%**
**Patient symptoms**	
Anxiety	79.3%
Fatigue	89.7%
Discomfort	89.7%
Include patient symptoms only if they lead to a return to bed	75.0%
**Other events**	
Increase in patient's body temperature/fever	86.2%
A mobilising patient tries to leave the ward	89.6%
Patient refusal to cooperate	86.2%
A mobilising patient disrupts other patients or their care	85.7%
Viewing another patient mobilise causes a patient to do something inappropriate or increases their agitation (e.g. they try to adjust their treatment)	82.7%
Classify as a) life-threatening event with serious risk of death; b) non-serious adverse event	17.2%^

**Excluded because they contradict with consensus agreement for inclusion of statement 'What counts as an unsafe change in physiological values? If it goes above/below a patient-specific target range'.*
***Excluded as this conflicts with consensus agreement for inclusion of (for lines and attachments) 'include if they are disrupted or dysfunctioning' and 'include if they are removed completely'.*
^Excluded as an adverse event classification has already reached consensus agreement for inclusion.
Percentage consensus is the total 'disagree' and 'strongly disagree'.

Supplementary Table 4: Adverse events that reached consensus for inclusion in the adverse event definition

Included adverse events	Percentage consensus
**Unsafe change in physiological observations**	
Bradycardia	89.3%
Tachycardia	75.0%
Hypotension	85.7%
Hypertension	82.1%
Arterial Oxygen Desaturation	85.2%
Tachypnoea	70.4%
Bradypnoea	89.3%
What counts as an unsafe change in physiological values?	
• If it stops the mobilisation treatment	77.8%
• If it does not resolve with rest	88.9%
• If it causes symptoms and/or haemodynamic instability	92.6%
• If it requires treatment	100.0%
• If it goes above/below a patient-specific target range	82.6%
**Airway**	
Any unplanned displacement of endotracheal tube or tracheostomy from original position placed	79.3%
Include the above if airway disrupted or dysfunctioning	93.1%
Include the above if airway removed completely	100.0%
Include the above if reintubation required	100.0%
**Cardiovascular**	
Syncope due to cardiovascular deterioration	93.1%
Myocardial infarction/ischaemia	92.6%
Any new arrhythmia	82.8%
Ignore arrhythmias that are not clinically concerning	93.1%
Include arrhythmias associated with symptoms and/or haemodynamic instability	92.5%
Include arrhythmias that do not resolve with rest	81.4%
Include arrhythmias that require treatment	96.3%
**Respiratory**	
Hypoxia and/or hypercapnia on arterial blood gases	73.9%
Unplanned increase in ventilator support	71.4%
**Neurological**	
Any neurological deterioration	86.2%
Include if neurological deterioration is persistent	89.6%
Include if neurological deterioration requires imaging /treatment.	86.2%
Seizure.	86.2%
Stroke	82.7%

**Lines and attachments**	
[Lines and attachments] Include if they are disrupted or dysfunctioning	75.8%
[Lines and attachments] Include if they are removed completely	96.5%
[Lines and attachments] Relevant tubes, drains, lines (e.g. if attached to organ support), cardiac devices should be specified separately on adverse event tool.	82.2%
**Falls and injuries**	
Any fall	79.3%
Include if fall was averted/ minimised by assistance of staff	79.3%
Include falls that cause physical injury	96.6%
Include falls that cause psychological injury	79.3%
Include falls requiring treatment	96.6%
The extent of the fall is important to state (e.g. fall to chair or the floor)	93.1%
Any injuries to patient	93.1%
Include if injury not recovered by 24 hours	89.6%
Include if injury delays other treatment plan	96.6%
Staff injuries related to patient mobilisation	82.7%
**Other events**	
Cardiac and/or respiratory arrest	100.0%
Death	96.5%
Embolisation of a thrombosis	89.3%
Any disruption to wounds / dressings / surgical incisions	72.4%
Requires acute surgery as a result of mobilisation.	96.6%
Changes to skin integrity	75.9%
Include adverse events if they do not resolve with rest.	93.1%
Include if mobilisation has to be stopped early due to an unsafe event	75.8%
If mobilisation leads to an increase in any additional organ support modalities	79.3%
The adverse event tool should state at the end: 'Include any other patient deterioration thought to be related to mobilisation'	75.9%
Classify as a) events that stop mobilisation; b) events that lead to further consequences other than stopping; c) events with serious consequences (increased length of stay, surgery, death)	72.4%

Percentage consensus is the total 'agree' and 'strongly agree' ratings.

Supplementary Table 5: Adverse events that did not reach consensus with a summary of round three responses

Adverse events that did not reach consensus	Percentage 'strongly disagree' + 'disagree'	Percentage 'undecided'	Percentage 'agree' + 'strongly agree'
**Unsafe change in physiological observations**			
For tachycardia only: Define using age-predicted maximal heart rate?	69.5%	13.0%	17.4%
**Cardiovascular**			
Any cardiovascular deterioration	65.5%	3.4%	31.0%
Dizziness due to cardiovascular deterioration	68.9%	24.1%	6.8%
Increased need for vasoactive drug support	17.2%	13.8%	68.9%
**Lines and attachments**			
Any unplanned movement of any indwelling devices, lines, tubes or drains.	51.7%	10.3%	37.9%
**Patient symptoms**			
Increased pain	69.0%	6.9%	24.1%
Agitation	62.1%	0%	37.9%
Patient distress (even if unable to fully communicate specific problem)	51.7%	6.9%	41.3%
**Other events**			
Bearing weight inappropriately on an injured leg	41.4%	10.3%	48.2%
Increase in patient hallucinations (e.g. to the extent they require restraint/sedation or they cause injury to themselves or the environment).	58.6%	3.4%	37.9%
Mobilising results in a prolonged recovery time: more than twice the duration of the mobilising period	69.0%	10.3%	20.7%

NB percentages sometimes do not exactly add up to 100 due to rounding.

Supplementary Table 6: Results of content analysis of participant comments made in rounds two and three for the adverse event definition

Adverse event	Round two comments	Round three comments
	**CARDIOVASCULAR EVENTS**	
Any cardiovascular deterioration	When defining a deterioration, consider that it is *abnormal* if heart rate and blood pressure does not change in response to exercise.	• Is the emphasis here on "Any" deterioration without any threshold (e.g. 1 mmHg of change in BP)?<br>• The comment from round two seems important and since we can't always have predictable responses of HR and BP in the ICU (and because some "exercise" in the ICU has shown to have very little effect on HR and BP) I am now completely changing my rating to strongly disagree.<br>• I disagree with the comment from round two, it is all depending on the level of exercise intensity.
Dizziness due to cardiovascular deterioration	Dizziness is not unusual, does not usually substantially impact upon the patient and settles if one takes things slowly.	
Increased need for vasoactive drug support	• An increase in vasoactive drug support can actually allow mobilisation to happen because it can be used to compensate for the usual decrease in blood pressure. So it is not necessarily an unsafe event that substantially impacts upon the patient.<br>• Only an unsafe event if in response to a heart/blood pressure deterioration or if increased above a certain amount.	• Based on the round two comments, I think we should be clarifying that the increased need for drug support is AFTER the start of mobilization in response to a deterioration rather than a proactive plan.<br>• An increased need for vasoactive drug support may be expected upon mobilisation, how much of an increase is significant is difficult to define and differs between drugs and patients.
Myocardial infarction/ischaemia	I think a specific diagnosis is not suitable for an unsafe event definition.	
General comments on arrhythmia	• Arrhythmias are not relevant as they are seldom related to mobilisation.<br>• It may be difficult for less experienced clinicians to decide what a clinically concerning arrhythmia is.<br>• Premature ventricular contractions (PVC) [a certain type of abnormal heart rhythm] may not be clinically concerning and may or may not be related to immobility.<br>• We all have sinus tachycardia (ST) [a fast heart rate] when we exercise so that should not be considered an unsafe event in the ICU.	

	• Atrial fibrillation [a certain type of abnormal heart rhythm] is common in ICU patients and it is often debatable whether mobilisation caused it or if it was going to happen anyway.<br>• Some clinically concerning abnormal heart rhythms may be symptom free and/or have maintained blood pressure, but should still be included as an unsafe event	
Any new arrhythmia		Rating changed to disagree as sinus tachycardia would fall into this definition and should NOT be considered an adverse event. Also, more appropriate definitions have already reached consensus.
	**RESPIRATORY EVENTS**	
General comments on respiratory events	• Some of these events may be very hard to define in order to measure as an unsafe event.<br>• Some of these events happen as a result of mobilisation but are not necessarily unsafe. Some are only unsafe if there is a clinical deterioration.<br>• Sometimes we want to push people a bit	The issue with all these adverse events is the response to them. Clearly if they are resolved quickly then there is no harm, but if they are not resolved then it could result in deterioration.
Extreme coughing	It is not unusual for patients who mobilise to experience coughing as a result of opening up previously collapsed parts of the lung. It is a beneficial effect!	
Large amounts of chest secretions	It is not unusual and it is beneficial to bring up phlegm during mobilisation as patients open up their lungs.	
Dyspnoea	Breathlessness can be a normal response to exercise and not necessarily an unsafe event. It depends on the extent.	
Increased work of breathing	Increased work of breathing can be a normal response to exercise and not necessarily an unsafe event. It depends on the extent.	
Hypoxia and/or hypercapnia on arterial blood gases	• Pulse oximetry is a more realistic way to define hypoxia as many patients don't have an arterial line.<br>• Depending on the underlying pulmonary pathology, hypoxia may be an expected result of exercise and may be managed with increased FiO_2.<br>• Hypercapnia is more problematic although very uncommon as a result of exercise.<br>• Hypercapnia and chronic hypoxia may be acceptable in patients such as those with COPD/long smoking history.	
Accidental disconnection from	• These events are rare and often trivial, e.g. if it is realised immediately and they are reconnected it may be fine.	• I agree with the round two comment; this is a bit vague and hard to understand so I revised my rating to disagree.

mechanical ventilator	• But it is potentially serious, so it important to know whether it lead to anything bad actually happening to the patient.	• The round two comment regarding whether it led to further negative events is important. Therefore it is not necessarily an adverse event in itself if it is realised immediately but provision for identification of further negative outcomes must be made
Ventilator dyssynchrony	• It is very uncommon for this to be significant in patients where the ventilator [breathing machine] has been adequately adjusted to the right settings.<br>• It depends on the extent.	• Ventilator dyssynchrony is associated with increased ICU/hospital length of stay, duration of mechanical ventilation and mortality. Significant dyssynchrony might be that which interferes with gas exchange or necessitates cessation of mobilisation.<br>• I have changed my rating to disagree based on the assumption that it is transient and the patient settles.
Unplanned increase in ventilator support	Increased ventilator support can be provided to allow the patient to mobilise.	
**ABNORMAL CHANGES IN PHYSIOLOGICAL PARAMETERS**		
General comments on abnormal changes in physiological parameters	• Changes in physiological values e.g. heart rate and blood pressure, can be a normal effect of exercise. This needs to be considered when deciding what is unsafe.<br>• Sometimes we want to push people a bit	
Tachycardia	Do not count tachycardia that is proportionate to exercise as unsafe	
Hypotension	It is not unusual for there to be a decrease in blood pressure during mobilisation because the body's normal reflexes have been effected.	
Arterial Oxygen Desaturation	A desaturation which does not resolve with rest or requires the patient to need increased oxygen support is an unsafe event.	
Bradypnoea	Why would a patient's breathing rate become too low during mobilisation? Bradypnoea must be incredibly uncommon.	
If it goes above/below a patient-specific target range it counts as an unsafe change in physiological values	Unsafe changes should be defined the same for all patients rather than a patient-specific target range as it is not practical to perform a calculation to measure an adverse event.	
For tachycardia only: Define using age-predicted maximal heart rate?	Age-predicted max. heart rate is not a feasible approach for implementation for clinical practice or research	
**NEUROLOGICAL EVENTS**		
General comments on	• I think it would be unlikely that the mobilisation would cause	

neurological events	neurological events – it would happen anyway.<br>• As long as no other obvious reasons like low blood sugar can explain it.<br>• There are some neurological deteriorations that are less significant	
Any neurological deterioration		I changed rating to disagree because I think it is unlikely to find many scenarios where mobilization CAUSED this event and the definition of this event is too vague to be reliably measured for clinical or research purposes.
Seizure and stroke	• A seizure and stroke are unlikely to be CAUSED by mobilization.<br>• Stroke caused by lack of blood supply to the brain not relevant unless there is another factor involved like atrial fibrillation [abnormal heart rhythm]. Would include a stroke caused by bleeding inside the skull as an unsafe event.	
**AIRWAY EVENTS**		
Any unplanned displacement of endotracheal tube or tracheostomy from original position placed	• Detecting *any* unplanned displacement would require, for instance, a chest X-ray before and after each mobilization session which would not be feasible.<br>• This should be compared to the amount of unplanned movement of the breathing tube the rest of the time that the patient is not mobilising.	
Include the above if airway disrupted or dysfunctioning	Any disruption of “life support system” could cause alarm to patient.	
**LINES AND ATTACHMENTS**		
General comments on lines and attachments	Not all attachments are of equal importance for instance in terms of the risk to the patient if they are removed. Only important ones should be included.	Given that we have reached consensus on more defined and measureable events in this category, the remaining events above are not needed – too vague or not helpful in light of those that we have reached consensus on.
Any unplanned movement of any indwelling devices, lines, tubes or drains		I changed my answer to disagree because what happened in consequence is important and “movement” is not suitable for high risk.
Include the above if they are pulled, trapped or tangled	• Tangling is less important than pulled or trapped.<br>• Tangled is more common with trips to the scanner than mobilisation.	

Relevant tubes, drains, lines (e.g. if attached to organ support), cardiac devices should be specified separately on adverse event tool.	• Not sure if they all need to be reported separately<br>• These should be separated by whether they are necessary to maintain life or not.	
**FALLS AND INJURIES**		
General comments on falls	• We should simply measure whether there was a fall or not.<br>• Severity of fall and consequential damage should be noted.	
Any falls		I changed my answer to disagree as “any fall” is not specific.
Include if fall was averted/ minimised by assistance of staff		I think this will be too difficult to reliably measure; and since we reached consensus on extend of the fall, we would already define a fall as controlled lowering to chair so this category around averted/minimized is redundant/vague/not needed
The extent of the fall is important to state	I would only include falls to the ground, not to the chair	
Staff injuries related to patient mobilisation	Staff injuries are important but seem to be a quite different area that would require more detailed discussion to accurately measure	This is vague and may be difficult to measure. If the patient is being repositioned in bed dependently and a member of staff has a back injury, it might be construed as an injury during mobilisation, but it is not related.
**PATIENT SYMPTOMS**		
General comments on patient symptoms	• These are weak definitions of unsafe events if they are just measured by clinician personal opinion of patient symptoms.<br>• Many of these symptoms will be present to some degree in many patients and can usually be prevented if the clinician takes actions and educate patients prior to mobilization.<br>• Only an unsafe event if they interfere with treatment or are caused by another unsafe event	• I changed my ratings to disagree on review of the helpful round two comments.<br>• Severe symptoms leading to cessation of mobilisation would appear significant
Increased pain; discomfort.	Pain and discomfort from trauma or operations need to be managed so that mobilisation can occur, but it is unrealistic to think that it will be absent.	
Agitation	Agitation usually improves with mobilisation.	
Fatigue	Fatique is a normal response to exercise and exercise usually helps with sleep. However one needs to be watchful for excessive fatigue due to inadequate rest between mobilisation sessions.	
Patient distress	Distress could be included even if it does not cause the patient to be	

	returned to bed.	
**OTHER EVENTS**		
Embolisation of a thrombosis	It would be difficult to know whether this has occurred during mobilisation without imaging and investigations.	
Any disruption to wounds / dressings / surgical incisions		We want to report on clinically important disruptions. The term 'any' would indicate a potentially trivial disruption to a small dressing and seems too extreme for reporting.
Bearing weight inappropriately on an injured leg	Agree only if it results in a fracture or the need for other treatment.	This is too vague – we need to include implications as it is only an event if there are negative implications.
Increase in patient's body temperature/fever	Not sure this could ever be caused by mobilisation	
Increase in patient hallucinations	• Not sure that mobilisation could cause this. This would be extremely unusual and usually there is a decrease in agitation with mobilisation.<br>• Hallucinations are difficult to evaluate in many non-verbal patients, so agitation may be a better term.	
A mobilising patient disrupts other patients or their care	This indicates inadequate supervision/health care rather than an adverse event of the mobilisation.	
Viewing another patient mobilise causes a patient to do something inappropriate or increases their agitation	This would be most likely in the context of inadequate nursing care/communication rather than an adverse event of the mobilisation.	When a patient has achieved some mobilisation or is aware that others have achieved that patient is then emboldened to attempt further solo excursions to the peril of all.
**GENERAL STATEMENTS**		
Comments on general statements		Some of these events that require additional medical input e.g. sorting dislodged lines, or physical deteriorations would need to be reported as a clinical incident and therefore must be an adverse event. Similarly, I do not think we should be trying to say that nothing is an adverse event.
Include adverse events if they do not resolve with rest.	This would not make sense with some of the events listed above e.g. a stroke.	This is too vague to be useful – what time period should be allowed for rest? We have many other general statements that reached consensus that were helpful, so we don't need this one.
If mobilisation leads to	Depends on what organ support e.g. for renal [kidney] replacement	

an increase in any additional organ support modalities	therapy the evidence is that filter life is improved by mobilisation.	
General comments on adverse event classifications	• Classification should be ‘adverse events’ and ‘serious adverse events’<br>• All adverse events should be correlated with the risk assessment of the mobilisation procedure.<br>• Is there a middle ground between these two definitions?	
Classify as a) events that stop mobilisation; b) events that lead to further consequences other than stopping; c) events with serious consequences (increased length of stay, surgery, death)	Should increased length of stay be considered as an event of serious consequence when compared to death or surgery?	
Classify as a) life-threatening event with serious risk of death; b) non-serious adverse event	This is too blunt to use since rarely is an event life threatening based on published data	

Examples of changes made to adverse event tool in response to user feedback

Examples of amalgamation of adverse events that reached consensus

- 'Hypoxia and/or hypercapnia on arterial blood gases' combined with 'Arterial oxygen desaturation'.
- 'Unplanned increase in ventilator support' given as an example of requiring treatment in response to an unsafe change in physiological observations.
- 'Changes to skin integrity' given as an example of 'any injuries to patient'.

General statements

Some general statements could not be used as overarching principles as they conflicted with other included events. Therefore, they were included at the end of the tool to cover any other adverse event not captured by the rest of the tool. For example:

- 'include adverse events if they do not resolve with rest'
- 'mobilisation leads to an increase in any additional organ support modalities'

Wording changes

For definitions of unsafe changes in physiological observations:

- 'if it requires treatment' changed to 'it requires a new treatment or a substantial increase in existing treatment'. This clarifies *a priori* meaning as otherwise it could be read to include insignificant changes to supplementary oxygen, or an increase in vasoactive drugs that had been excluded from the tool.
- 'if it goes above/below a patient-specific target range' changed to 'a patient-specific target range for an adverse event'. Testing feedback highlighted confusion of 'target rage' with the daily goal for the patient rather than a pre-defined target range for unsafe occurrences during mobility.

- 'If mobilisation leads to an increase in any additional organ support modalities' became 'starting new organ support modalities' otherwise it would include an increase in vasoactive drugs (which had been excluded from the tool).

Supplementary Table 7: Vasoactive drugs risk assessment tool results of content analysis of round one

**Round 1 results**
**General statements**
What should be considered when judging the risk of mobilising patients receiving vasoactive drugs?<br>• Vasoactive drug dose<br>• Recent change in vasoactive drug dose<br>• Number of vasoactive drugs<br>• Particular types of vasoactive drugs<br>• Reason for vasoactive drug use<br>• Type of venous access (e.g. peripheral/route of central access)<br>• Recent trends in heart rate and blood pressure<br>• Heart rhythm<br>• Signs of inadequate perfusion (e.g. lactate, central venous oxygen saturation)<br>• Echo findings<br>• Disease specific factors<br>• Premorbid functional status<br>• Unit culture and resources<br>• Degree of cooperativeness of patient<br>For vasoactive drug dose:<br>• Specific threshold doses should be specified for low, higher and contraindicated groups<br>• Doses should just be considered as 'low', 'medium' or "high"<br>• Unable to specify cut off doses for these different levels of risk, since there are multiple issues that must be considered at the same time.<br>For heart rate and blood pressure:<br>• Specify using specific thresholds for the low, higher risk and contraindicated groups.<br>• Target ranges would vary according to specific patient characteristics.<br>Risk should be judged using a stepwise approach to mobilisation: progression to the next level occurring if vital signs remain stable.<br>No patient is low risk mobilising on a vasoactive agent.<br>All out of bed mobilisation contraindicated for patients receiving vasoactive drugs.<br>No specific cut off thresholds between low and high risk, since there are multiple issues that must be considered at the same time.
**Indicators that a patient has changed from low risk to higher risk or that mobilisation is contraindicated.**
**Vasoactive drug specific indicators of risk**
Vasoactive drugs used for patient pathology rather than counteracting other treatments e.g. epidural<br>Unsecure central venous access or with possibility of occluding/disconnecting line during mobilisation<br>DOSES THAT ARE REQUIRED TO MAINTAIN CARDIOVASCULAR STABILITY:<br>• Low dose vasoactive drugs<br>• Medium dose of vasoactive drugs<br>• Higher dose of vasoactive drugs<br>• Low dose vasoactive drugs with a primary inotropic effect<br>• Medium or high doses of vasoactive drugs with a primary inotropic effect<br>• No recent increase in dose<br>• Any recent increase in dose<br>• Dose needs to be increased during mobilisation<br>• Single vasoactive drug<br>• Two vasoactive drugs<br>• More than two vasoactive drugs<br>• Noradrenaline/norepinephrine dose < 0.1 mcg/kg/min*<br>• Noradrenaline/norepinephrine dose between 0.1-0.2 mcg/kg/min*<br>• Noradrenaline/norepinephrine dose 0.2-0.5 mcg/kg/min*

- Noradrenaline/norepinephrine dose > 0.5 mcg/kg/min*
- Adrenaline/epinephrine dose < 0.1 mcg/kg/min*
- Adrenaline/epinephrine dose between 0.1-0.2 mcg/kg/min*
- Adrenaline/epinephrine dose 0.2-0.5 mcg/kg/min*
- Adrenaline/epinephrine dose > 0.5 mcg/kg/min*
- Dobutamine dose < 5 mcg/kg/min*
- Dobutamine dose 5-10 mcg/kg/min*
- Dobutamine dose > 10 mcg/kg/min*
- Any dose adrenaline/epinephrine
- Any dose of dobutamine
- Any dose of dopamine
- Any dose of enoximone
- Any dose of isoprenaline
- Any dose of levosimendan
- Any dose of noradrenaline/norepinephrine
- Any dose of meteraminol
- Any dose of milrinone
- Any dose of vasopressin

*drug dose assumes a typical weight of 70kg, therefore please multiply by 70 to convert to mcg/min.

**Cardiovascular indicators of risk**
Failure to achieve satisfactory cardiovascular stability on any dose of vasoactive drugs
Cardiovascular instability: difficulty in achieving targets
Cardiovascular instability causing symptomatic changes.
Cardiovascular instability during recent mobilisation/change in posture
Cardiovascular instability during any adjustment of vasoactive drug
Any Hypotension/Hypertension
Uncontrolled, symptomatic hypotension/ hypertension
Blood pressure below target range despite escalating support
Any tachycardia /bradycardia
New/recent onset arrhythmia with no haemodynamic compromise
New/recent onset of arrhythmias with hemodynamic instability
Arrhythmia that occurred >24 hours ago
Patient receiving anti-arrhythmic medication
Active requirement of fluid boluses
Signs of hypovolaemia/inadequate perfusion
Impaired cardiac output but pre-existing low cardiac output state
Evidence of impaired cardiac output
Any recent/current signs of myocardial ischemia/infarction
Increase in cardiothoracic ratio on chest x-ray film
Post-cardiac arrest
ECHO findings of submassive pulmonary embolus with right heart strain
Massive pulmonary emboli
Cardiovascular co-morbidities

**Other indicators of risk**
Any first mobilisation treatment on a vasopressor
Previous adverse events during mobilization
Patient in an acute phase of critical illness
Patient presents with an unclear diagnosis/ uncertain clinical course
Patients who are slowly improving from an episode of critical illness
Patient frailty
Patients receiving palliative care
First mobilisation post-surgery or after prolonged bed rest
If mobilization has been specifically limited post-surgery (e.g. open abdomen) or because of injuries
Inadequate analgesia

Autonomic dysregulation
Severe dyspnoea
Presence of an endotracheal tube
Requiring mechanical ventilation
Higher ventilation support required e.g. higher FiO2 and PEEP requirements
Severe respiratory failure e.g. requiring deep sedation +/- paralysis and lung protective ventilation
Renal replacement therapy as long as not femoral access
Renal replacement therapy with femoral access
ECMO
Intra-aortic balloon pump
Percutaneous cardiopulmonary support (e.g. pacing)
Patient confusion/ delirium
Reduced conscious level
Obesity
Stroke
Recent episodes of pulmonary oedema
Recently active ischemia to limb (past 4-6 hours) now currently stable
Diagnosis of acute aortic dissection
pH acidotic on Blood Gas Analysis
Active organ ischemia that is new or acute (less than 6 hours)
Active cooling
Active bleeding
Unstable fractures or spinal injuries
Staff inexperience

Supplementary Table 8: Vasoactive drugs risk assessment tool example of round one content analysis process

Original coding	Interim categories	Final wider category classifications
• 0.05 mcg/kg/min of Norepinephrine<br>• norepinephrine 0.8mcg/kg/min<br>• Noradrenaline 0.1-0.2mg/kg/min<br>• Noradrenaline 0.15mcg/kg/min to 0.2mcg/kg/min<br>• Noradrenaline 0.1 mic/kg/min<br>• Noradrenaline 0.2mcg/kg/min<br>• norepinephrine 0.8mcg/kg/min<br>• Noradrenaline 5mcg/min<br>• Noradrenaline 0-10 mcg/min<br>• Norad 5 Mcg/min<br>• Norad 5-10mcg<br>• Noradrenaline 10-20 mcg/min<br>• noradrenaline 10mcg/min<br>• Noradrenaline 11-29mcg/min<br>• Noradrenaline 20 mcg/min<br>• Noradrenaline 30 mcg/min<br>• 10 mg norepinephrine/hr.<br>• Noradrenaline: Near maximal doses<br>• 3 mg Noradrenalin < 6ml/h	• 0.01 mcg/kg/min<br>• 0.05 mcg/kg/min<br>• 0.07 mcg/kg/min<br>• 0.09mcg/kg/min<br>• 0.07 – 0.14 mcg/kg/min<br>• 0.14 mcg/kg/min<br>• 0.14 – 0.29 mcg/kg/min<br>• 0.1-0.2mg/kg/min<br>• 0.1 mcg/kg/min<br>• 0.15mcg/kg/min to 0.2mcg/kg/min<br>• 0.16 – 0.41mcg/kg/min<br>• 0.2mcg/kg/min<br>• 0.29 mcg/kg/min<br>• 0.43 mcg/kg/min<br>• 0.8mcg/kg/min	• Any dose of noradrenaline/norepinephrine<br>• Noradrenaline/norepinephrine dose < 0.1 mcg/kg/min*<br>• Noradrenaline/norepinephrine dose between 0.1-0.2 mcg/kg/min*<br>• Noradrenaline/norepinephrine dose 0.2-0.5 mcg/kg/min*<br>• Noradrenaline/norepinephrine dose > 0.5 mcg/kg/min*<br>*drug dose assumes a typical weight of 70kg, therefore please multiply by 70 to convert to mcg/min.

Supplementary Table 9: Indicators of risk that reached consensus for exclusion from the risk assessment tool

**Excluded adverse events**	**Percentage consensus**
**General statements**	
What should be considered when judging the risk of mobilising patients receiving vasoactive drugs?	
• Type of venous access (e.g. peripheral/route of central access)	81.8%
• Unit culture and resources	72.7%
For heart rate and blood pressure: Specify using specific thresholds for the low, higher risk and contraindicated groups.	39.1%*
No patient is low risk mobilising on a vasoactive agent	81.8%
All out of bed mobilisation contraindicated for patients receiving vasoactive drugs	100%
**Indicators that a patient has changed from low risk to higher risk or that mobilisation is contraindicated**	
**Vasoactive drug specific indicators of risk**	
DOSES THAT ARE REQUIRED TO MAINTAIN CARDIOVASCULAR STABILITY:	
Low dose vasoactive drugs	85.8%
Low dose vasoactive drugs with a primary inotropic effect	77.2%
No recent increase in dose	86.4%
Single vasoactive drug	86.4%
Noradrenaline/norepinephrine dose < 0.1 mcg/kg/min	72.7%
Any dose adrenaline/epinephrine	81.8%
Any dose of dobutamine	81.8%
Any dose of dopamine	81.8%
Any dose of noradrenaline/norepinephrine	86.3%
**Cardiovascular indicators of risk**	
Any Hypotension/Hypertension	72.7%
Any tachycardia /bradycardia	77.3%
Arrhythmia that occurred >24 hours ago	78.3%
Patient receiving anti-arrhythmic medication	82.6%
Cardiovascular co-morbidities	90.9%
**Other indicators of risk**	
Patient presents with an unclear diagnosis/ uncertain clinical course	77.2%
Patients who are slowly improving from an episode of critical illness	91.3%
Patient frailty	95.4%
Patients receiving palliative care	90.9%
First mobilisation post-surgery or after prolonged bed rest	86.4%
Presence of an endotracheal tube	77.2%
Requiring mechanical ventilation	77.2%
Renal replacement therapy as long as not femoral access	81.8%
Obesity	73.9%

Percentage consensus is the total 'disagree' and 'strongly disagree'.
**Excluded after round two as it contradicts statement that had reached consensus for inclusion 'For heart rate and blood pressure: Target ranges would vary according to specific patient characteristics'.*

Supplementary Table 10: Indicators of risk that reached consensus for inclusion in the risk assessment tool

Included indicators of risk	Percentage consensus
**General statements**	
What should be considered when judging the risk of mobilising patients receiving vasoactive drugs?	
• Vasoactive drug dose	91.3%
• Recent change in vasoactive drug dose	95.7%
• Number of vasoactive drugs	78.3%
• Particular types of vasoactive drugs	90.9%
• Reason for vasoactive drug use	90.9%
• Recent trends in heart rate and blood pressure	95.6%
• Heart rhythm	78.2%
• Signs of inadequate perfusion (e.g. lactate, central venous oxygen saturation)	82.6%
• Disease specific factors	78.3%
• Premorbid functional status	86.4%
• Degree of cooperativeness of patient	81.8%
For vasoactive drug dose: Unable to specify cut off doses for these different levels of risk, since there are multiple issues that must be considered at the same time.	95.4%
For heart rate and blood pressure: Target ranges would vary according to specific patient characteristics	87.0%
Risk should be judged using a stepwise approach to mobilisation: progression to the next level occurring if vital signs remain stable.	87.0%
**Indicators that a patient has changed from low risk to higher risk**	
**Vasoactive drug specific indicators of risk**	
Vasoactive drugs used for patient pathology rather than counteracting other treatments e.g. epidural	81.8%
DOSES THAT ARE REQUIRED TO MAINTAIN CARDIOVASCULAR STABILITY:	
Medium dose of vasoactive drugs	86.3%
Medium or high doses of vasoactive drugs with a primary inotropic effect	72.7%
Any recent increase in dose	81.8%
Dose needs to be increased during mobilisation	81.8%
Two vasoactive drugs	90.9%
More than two vasoactive drugs	77.3%
Noradrenaline/norepinephrine dose 0.2-0.5 mcg/kg/min	72.7%
**Cardiovascular indicators of risk**	
Cardiovascular instability: difficulty in achieving targets	77.2%
Cardiovascular instability causing symptomatic changes.	78.2%
Cardiovascular instability during recent mobilisation/change in posture	73.9%
Cardiovascular instability during any adjustment of vasoactive drug	86.4%
New/recent onset arrhythmia with no haemodynamic compromise	77.3%
New/recent onset of arrhythmias with hemodynamic instability	90.9%

Signs of hypovolaemia/inadequate perfusion	72.7%
Evidence of impaired cardiac output	77.3%
**Other indicators of risk**	
Any first mobilisation treatment on a vasopressor	77.3%
Previous adverse events during mobilization	86.9%
If mobilization has been specifically limited post-surgery (e.g. open abdomen) or because of injuries	81.8%
Inadequate analgesia	72.7%
Autonomic dysregulation	78.3%
Severe dyspnoea	86.4%
Higher ventilation support required e.g. higher FiO2 and PEEP requirements	73.9%
ECMO	72.7%
Reduced conscious level	72.7%
Active organ ischemia that is new or acute (less than 6 hours)	77.2%
Staff inexperience	81.8%
**Indicators that mobilisation is contraindicated.**	
**Vasoactive drug specific indicators of risk**	
Adrenaline/epinephrine dose > 0.5 mcg/kg/min required to maintain cardiovascular stability	86.4%
**Cardiovascular indicators of risk**	
Failure to achieve satisfactory cardiovascular stability on any dose of vasoactive drugs	95.5%
ECHO findings of submassive pulmonary embolus with right heart strain	72.7%
Massive pulmonary emboli	81.0%
**Other indicators of risk**	
Severe respiratory failure e.g. requiring deep sedation +/- paralysis and lung protective ventilation	86.4%
Diagnosis of acute aortic dissection	72.7%
Active cooling	86.4%
Active bleeding	86.4%
Unstable fractures or spinal injuries	86.4%

Percentage consensus is the total ‘agree’ and ‘strongly agree’ ratings. The exception to this is indicators that mobilisation is contraindicated where percentage consensus is just the total who voted ‘contraindicated’.

Supplementary Table 11: Indicators of risk that did not reach consensus for the risk assessment tool, with a summary of round three responses

Indicators of risk that did not reach consensus	strongly disagree and disagree	un-decided	agree and strongly agree	Or, contra-indicated
**General statements**				
What should be considered when judging the risk of mobilising patients receiving vasoactive drugs?				
• Echo findings	36.4%	4.5%	59.1%	
For vasoactive drug dose:				
• Specific threshold doses should be specified for low, higher and contraindicated groups	22.7%	9.1%	68.2%	
• Doses should just be considered as 'low', 'medium' or "high"	59.0%	4.5%	36.4%	
No specific cut off thresholds between low and high risk, since there are multiple issues that must be considered at the same time.	31.8%	36.4%	31.8%	
**Indicators that a patient has changed from low risk to higher risk or that mobilisation is contraindicated**				
**Vasoactive drug specific indicators of risk**				
Unsecure central venous access or with possibility of occluding/disconnecting line during mobilisation*	9.1%	0%	63.6%	27.3%
DOSES THAT ARE REQUIRED TO MAINTAIN CARDIOVASCULAR STABILITY:				
Higher dose of vasoactive drugs*	0%	4.5%	68.2%	27.3%
Noradrenaline/norepinephrine dose between 0.1-0.2 mcg/kg/min	22.7%	54.5%	18.1%	4.5%
Noradrenaline/norepinephrine dose > 0.5 mcg/kg/min*	0%	13.6%	18.2%	68.2%
Adrenaline/epinephrine dose < 0.1 mcg/kg/min	59.0%	31.8%	9.1%	0%
Adrenaline/epinephrine dose between 0.1-0.2 mcg/kg/min	18.2%	50.0%	22.7%	9.1%
Adrenaline/epinephrine dose 0.2-0.5 mcg/kg/min*	0%	9.1%	45.4%	45.5%
Dobutamine dose < 5 mcg/kg/min	68.1%	13.6%	18.1%	0%
Dobutamine dose 5-10 mcg/kg/min	19.0%	42.9%	33.4%	4.8%
Dobutamine dose > 10 mcg/kg/min*	4.5%	18.2%	18.1%	59.1%
Any dose of enoximone	31.8%	54.5%	9.0%	4.5%
Any dose of isoprenaline	36.3%	45.5%	18.2%	0%
Any dose of levosimendan	45.4%	40.9%	13.6%	0%
Any dose of meteraminol	63.6%	18.2%	18.2%	0%
Any dose of milrinone	50.0%	27.3%	22.7%	0%
Any dose of vasopressin	54.5%	13.6%	31.8%	0%
**Cardiovascular indicators of risk**				
Uncontrolled, symptomatic hypotension/ hypertension*	0%	0%	31.8%	68.2%
Blood pressure below target range despite escalating support*	4.8%	0%	47.6%	47.6%
Active requirement of fluid boluses	54.5%	9.1%	31.8%	4.5%
Impaired cardiac output but pre-existing low cardiac output state	27.3%	4.5%	63.6%	4.5%

Any recent/current signs of myocardial ischemia/infarction*	0%	0%	31.8%	68.2%
Increase in cardiothoracic ratio on chest x-ray film	54.5%	45.5%	0%	0%
Post-cardiac arrest	54.5%	13.6%	9.1%	22.7%
**Other indicators of risk**				
Patient in an acute phase of critical illness	63.6%	13.6%	18.2%	4.5%
Renal replacement therapy with femoral access	59.0%	18.2%	13.6%	9.1%
Intra-aortic balloon pump*	4.5%	0%	27.3%	68.2%
Percutaneous cardiopulmonary support (e.g. pacing)*	13.6%	0%	59.1%	27.3%
Patient confusion/ delirium	36.4%	9.1%	54.5%	0%
Stroke	68.1%	13.6%	18.2%	0%
Recent episodes of pulmonary oedema	59.1%	18.2%	18.2%	4.5%
Recently active ischemia to limb (past 4-6 hours) now currently stable	40.9%	0%	50.0%	9.1%
pH acidotic on Blood Gas Analysis*	13.6%	13.6%	68.2%	4.5%

*Indicators of risk that were eventually included in the risk tool because the total of ‘agree’ and ‘strongly agree’ plus the total for ‘contraindicated’ reaches the consensus threshold for inclusion.
NB percentages sometimes do not exactly add up to 100 due to rounding.

Supplementary Table 12: Undecided indicators of risk that were included as votes for ‘agree’ plus ‘strongly agree’ plus ‘contraindicated’ were greater than or equal to 70%

Unsecure central venous access or with possibility of occluding/disconnecting line during mobilisation
Higher dose of vasoactive drugs
Noradrenaline/norepinephrine dose > 0.5 mcg/kg/min
Adrenaline/epinephrine dose 0.2-0.5
Dobutamine dose > 10 mcg/kg/min
Uncontrolled, symptomatic hypotension/ hypertension
Blood pressure below target range despite escalating support
Any recent/current signs of myocardial ischemia/infarction
Intra-aortic balloon pump
Percutaneous cardiopulmonary support (e.g. pacing)
pH acidotic on Blood Gas Analysis

Supplementary Table 13: Results of content analysis of participant comments made in rounds two and three for the risk assessment tool

Indicator of risk	Round two comments	Round three comments
**GENERAL STATEMENTS ON WHAT SHOULD BE CONSIDERED WHEN JUDGING THE RISK OF MOBILISING PATIENTS RECEIVING VASOACTIVE DRUGS**		
Comments on general statements	• Often safer to mobilise on vasoactive agent than off it, as easier to titrate up to compensate for usual loss of orthostatic reflexes.<br>• Combination with cardiac deterioration (especially cardiac output and arrhythmia) is important here	
Type of venous access	I can't imagine a case with a patient on a vasoactive agent without central access.	Many ICUs will give a more diluted concentration of vasoactive agents and provide in low doses via a peripheral IV; and occasionally vasoactive drugs need to be run via continuous dialysis access (in a patient with difficult access) and disruption of blood flow with mobility and continuous dialysis can disrupt flow of vasopressors; hence, type of access is important in this setting.
Echo findings		I believe echo findings are important, when available, to understand both left and right heart function, volume status, and any RV strain in the setting of vasopressors for submassive pulmonary embolus.
Unit culture and resources	Resources yes, unit culture no	Agreed - don't mobilise a high risk patient during a break time if insufficient staffing or insufficient senior staff available
Vasoactive drug doses should just be considered as 'low', 'medium' or "high"	I feel a classification of 'low', 'medium' or "high" is unclear.	This is too unclear to be useful; we are trying to simplify complex clinical decision-making too simplistically and are open to misinterpretation or ignoring other factors if the dose is "low".
All out of bed mobilisation contraindicated for patients receiving vasoactive drugs	Low dose vasopressor to counteract epidural related sympathetic blockade not a problem	
**VASOACTIVE DRUG SPECIFIC INDICATORS OF RISK**		
General comment of vasoactive drug specific indicators of risk	Answers based on patient severity for vasoactive drug selection	• Vasoactive drugs that are only used rarely would be a consideration for mobilisation.<br>• It is necessary to give the specific dose for 'medium' and 'higher' dose of vasoactive drugs.<br>• In my country noradrenaline and adrenaline are used

		for patients with severe circulatory instability and would be judged to be high risk.<br>• The problem with 'any dose' is that perhaps a patient could be mobilised with a low dose of the medication but absolutely not with a high dose.<br>• I am also concerned about having specific cut off values for each one of the drugs considering that a large number of clinicians may not have a good understanding of the drugs. In addition, the numbers can be confusing since they are different for each drug.
Unsecure central venous access or with possibility of occluding/disconnecting line during mobilisation	• I would have thought secure central venous access was a standard of care.<br>• The available evidence suggests that theoretical concerns regarding kinking with mobilisation out of bed (e.g. femoral site) are unfounded.	
Any recent increase in dose		My concern with the use of 'any' is that this could be a trivial change in dose.
Noradrenaline/norepinephrine dose 0.2-0.5 mcg/kg/min	Probably would not mobilise someone on >0.3 mcg/kg/min Noradrenaline out of bed.	
Adrenaline/epinephrine dose 0.2-0.5 mcg/kg/min	Probably would not mobilise someone on >0.3 mcg/kg/min Adrenaline out of bed.	
Any dose of levosimendan	With Levosimendan it depends on the indication.	
Any dose of milrinone	Tachycardia/tachyarrhythmia's can be problematic with Milrinone (independent of any mobilisation), especially if one uses a loading dose.	
**CARDIOVASCULAR INDICATORS OF RISK**		
General comments on cardiovascular indicators of risk	For some of these it depends on the situation or other factors not specified here	
Cardiovascular instability during recent mobilisation/change in posture; or during any adjustment of vasoactive drug	Depends on what the cardiovascular instability was.	
Any		The use of 'any' here is too vague - a 1 mmHg change

hypotension/hypertension		could be included.
New/recent onset arrhythmia with no haemodynamic compromise		Not all arrhythmias are the same – for instance, PVC would be less worrisome than rapid atrial fibrillation.
Active requirement of fluid boluses		The amount of fluid, the actual blood pressure and the responsiveness of blood pressure to fluid are all relevant here; can't make decision about this issue in isolation.
Signs of hypovolaemia/inadequate perfusion		• Inadequate perfusion is a different issue from hypovolemia. There can be signs of hypovolemia (e.g. a low jugular venous pressure) without clinically important hypotension or clinical consequences.<br>• Some clinical markers of inadequate perfusion (e.g. lactate) may be much more important than others (e.g. slow capillary refill time).
Impaired cardiac output but pre-existing low cardiac output state and evidence of impaired cardiac output		More clinical information regarding current state of patient would be needed for decision making here.
Any recent/current signs of myocardial ischemia/infarction	Depends how recent the MI is	
Post-cardiac arrest		The timing of arrest and the reason, if known, are important factors – was the arrest 1 week ago with stability since that time and a known reason for arrest that has resolved (e.g. hyperkalaemia)?
**OTHER INDICATORS OF RISK**		
General comments on other indicators of risk	Whilst many increase risk, it would not prevent mobilisation…just requires more planning	
Previous adverse events during mobilization	Depends on what the previous adverse event was as to whether mobilisation contraindicated.	
First mobilisation post-surgery or after prolonged bed rest		…sitting up can be higher risk; there are published data showing more potential safety events in this clinical scenarios
Autonomic dysregulation	Disagree as we should judge with specific rate of blood pressure.	
Severe respiratory failure e.g.		Paralysis is a separate issue that needs separate voting.

requiring deep sedation +/- paralysis and lung protective ventilation		
Renal replacement therapy as long as not femoral access; Renal replacement therapy with femoral access	The available evidence does not demonstrate any issues with renal replacement therapy and femoral access, and suggests improved filter life.	This would be more clear if continuous vs. intermittent Renal replacement therapy was specified
Intra-aortic balloon pump	Location affects feasibility and safety of some mobilization (femoral vs. axillary access)	
Percutaneous cardiopulmonary support	Contraindicated if full dependence on temporary pacing wire	
Patient confusion/ delirium		This would be more clear if it specified hyperactive vs. hypoactive.
Reduced conscious level	Drowsy patients can be mobilised out of bed which often improves drowsiness, but mobilising is more difficult if the patient is unconscious.	
Stroke	The AVERT study suggests that intensive very early mobilisation may be associated with worse outcomes. We probably need a dose finding/timing/titration study.	
Recent episodes of pulmonary oedema	It depends on the nature of the episodes and likely underlying cause.	
Diagnosis of acute aortic dissection		The extent of dissection and any surgical or intravascular management plan and guidance regarding blood pressure control is needed to make a decision. Many patients have dissection that is not acute or is acute with medical management only and would have less impact upon mobilisation.
pH acidotic on blood gas analysis		Need to specify severity and reason; pH 7.34 is acidotic but not a concern; acidosis from high chloride due to large volume of normal saline would be less concern than due to lactate;
Active cooling		Further clarification required as the indication for cooling may play a role – e.g. post cardiac arrest, hyperthermia or just a fever?
Active bleeding		Need to specify the source, control, location and severity of bleeding.

Unstable fractures or spinal injuries		The immobilization plan, location of fracture/injury and the management plan plays a role; there can be mobilization of unstable fracture of arm which may be easier than with leg.
Staff inexperience	This is a staff factor and is not suitable for a patient's risk judgement.	

Examples of changes made to risk assessment tool in response to user feedback

Examples of combining risk indicators that reached consensus

- ‘medium dose’ and ‘higher dose’ became ‘medium and above’.
- ‘New/recent onset arrhythmia with no haemodynamic compromise’ and ‘New/recent onset of arrhythmias with hemodynamic instability’ became ‘Recent onset arrhythmia with or without haemodynamic instability’.
- ‘ECHO findings of submassive pulmonary embolus with right heart strain’ combined with ‘Massive pulmonary emboli’ to result in ‘Submassive or massive pulmonary embolus’.
- ‘Uncontrolled, symptomatic hypotension/ hypertension’ covered by other included definitions of cardiovascular instability.
- ‘cardiovascular instability during any adjustment of vasoactive drug’ covered by ‘difficulty achieving targets’ and ‘recent increase in vasoactive drug dose’.

Example of wording clarification

- ‘diagnosis of acute aortic dissection’ became ‘untreated acute aortic dissection’

Supplementary Figure 1: Risk assessment tool initial summary page

Do you want to mobilise a patient out of bed receiving vasoactive drugs?

Principles

1. Assess risk using a stepwise approach: only progress mobilisation if vital signs remain stable.
2. Specific doses of vasoactive drugs to indicate when risk changes cannot be specified - dose should be taken within the context of individual patient risk factors.
3. The heart rate and blood pressure for different levels of risk will vary according to specific patient characteristics.
4. This tool is not designed for brain injured patients with specific haemodynamic targets.

Please consider:

Vasoactive drug specific

- Dose and recent change in dose
- Number and particular types of vasoactive drugs.
- Reason for use.

Cardiovascular

- Recent trends in heart rate and blood pressure.
- Heart rhythm.
- Signs of inadequate perfusion.

Other indicators

- Disease specific factors
- Premorbid functional status
- Degree of cooperativeness of patient

STOP: mobilisation is CONTRAINDICATED (IF YES, TURN OVER FOR MORE DETAILS)

- *Failure* to achieve satisfactory cardiovascular stability on any dose of vasoactive drugs?
- Pulmonary embolus?
- Aortic dissection?

Other things to consider: Severe respiratory failure? Active cooling? Active bleeding? Unstable fractures or spinal injuries?

TALK TO THE LEAD/SENIOR CLINICIAN BEFORE MOBILISATION: Caution because mobilisation has become HIGHER risk (IF YES, TURN OVER FOR MORE DETAILS)

Vasoactive drug specific

- Two or more vasoactive drugs?
- Medium dose of vasoactive drugs or above?
- Any recent increase in dose?
- Unsecure central venous access?
- Vasoactive drugs used for patient pathology rather than counteracting other treatments such as an epidural?

Cardiovascular

- Cardiovascular instability?
- Recent onset arrhythmia?
- Hypovolaemia/inadequate perfusion?
- Impaired cardiac output?
- Percutaneous cardiopulmonary support (e.g. pacing)?
- Myocardial ischemia/infarction?

Examples of other things to consider

- Staff inexperience?
- Patient's first mobilisation treatment on a vasopressor? Previous adverse events during mobilization?
- Acidosis?
- Severe dyspnoea? Higher ventilation support?
- Autonomic dysregulation?
- Active organ ischemia?
- ECMO / Intra-aortic balloon pump?
- Inadequate analgesia? If mobilization has been specifically limited post-surgery (e.g. open abdomen) or because of injuries?
- Reduced conscious level?

There may be other things in your environment that will add to risk.

Please note that this is a guidance tool only. When judging the risk of mobilising, the main priority is to evaluate the patient on an individual basis, with the assessment of the clinical team based on the context of specific patient circumstances outweighing the above principles.
